# A molecular brake that modulates spliceosome pausing at detained introns contributes to neurodegeneration

**DOI:** 10.1093/procel/pwac008

**Published:** 2022-11-11

**Authors:** Dawei Meng, Qian Zheng, Xue Zhang, Xuejiao Piao, Li Luo, Yichang Jia

**Affiliations:** Tsinghua-Peking Joint Center for Life Sciences, Tsinghua University, Beijing 100084, China; School of Life Sciences, Tsinghua University, Beijing 100084, China; School of Medicine, Tsinghua University, Beijing 100084, China; IDG/McGovern Institute for Brain Research, Tsinghua University, Beijing 100084, China; Tsinghua-Peking Joint Center for Life Sciences, Tsinghua University, Beijing 100084, China; School of Life Sciences, Tsinghua University, Beijing 100084, China; School of Medicine, Tsinghua University, Beijing 100084, China; IDG/McGovern Institute for Brain Research, Tsinghua University, Beijing 100084, China; Tsinghua-Peking Joint Center for Life Sciences, Tsinghua University, Beijing 100084, China; School of Medicine, Tsinghua University, Beijing 100084, China; IDG/McGovern Institute for Brain Research, Tsinghua University, Beijing 100084, China; Tsinghua-Peking Joint Center for Life Sciences, Tsinghua University, Beijing 100084, China; School of Medicine, Tsinghua University, Beijing 100084, China; Tsinghua Laboratory of Brain and Intelligence, Tsinghua University, Beijing 100084, China; Tsinghua-Peking Joint Center for Life Sciences, Tsinghua University, Beijing 100084, China; School of Medicine, Tsinghua University, Beijing 100084, China; IDG/McGovern Institute for Brain Research, Tsinghua University, Beijing 100084, China; Tsinghua Laboratory of Brain and Intelligence, Tsinghua University, Beijing 100084, China

**Keywords:** SNIP1, RNPS1, spliceosome, detained intron, neurodegeneration

## Abstract

Emerging evidence suggests that intron-detaining transcripts (IDTs) are a nucleus-detained and polyadenylated mRNA pool for cell to quickly and effectively respond to environmental stimuli and stress. However, the underlying mechanisms of detained intron (DI) splicing are still largely unknown. Here, we suggest that post-transcriptional DI splicing is paused at the B^act^ state, an active spliceosome but not catalytically primed, which depends on Smad Nuclear Interacting Protein 1 (SNIP1) and RNPS1 (a serine-rich RNA binding protein) interaction. RNPS1 and B^act^ components preferentially dock at DIs and the RNPS1 docking is sufficient to trigger spliceosome pausing. Haploinsufficiency of *Snip1* attenuates neurodegeneration and globally rescues IDT accumulation caused by a previously reported mutant U2 snRNA, a basal spliceosomal component. *Snip1* conditional knockout in the cerebellum decreases DI splicing efficiency and causes neurodegeneration. Therefore, we suggest that SNIP1 and RNPS1 form a molecular brake to promote spliceosome pausing, and that its misregulation contributes to neurodegeneration.

## Introduction

Introns are spliced from eukaryotic messenger RNA precursors (pre-mRNA) by the spliceosome via two transesterification reactions—branching and exon ligation (Padgett et al*.,* 1986). During these reactions, the spliceosome undergoes structural and compositional dynamics (Wahl et al., 2009; Shi, 2017; Wilkinson et al., 2020). Firstly, the 5ʹ splice site (SS), branch site (BS), and 3ʹ SS of an intron are recognized by the U1 small nuclear RNA (snRNA), SF1, and U2AF, respectively (E complex). Then, U2 small nuclear ribonucleoprotein particles (snRNPs) are recruited by the E complex to the BS (A complex), which then binds to U4/U6.U5 tri-snRNP to form the fully assembled and pre-catalytic spliceosome (B complex). The resulting B complex is empowered by an ATPase/helicase Brr2, remodeling to the active spliceosome (B^act^). Through additional remodeling by the ATPase/helicase Prp2, B^act^ matures into a catalytically activated spliceosome (B*), in which the branching reaction occurs. During the B-to-B^act^ and B^act^-to-B* transitions, a number of proteins are loaded/unloaded into the spliceosome, including more than 10 proteins recruited into early B^act^ and release of SF3a, SF3b, and pre-mRNA REtention and Splicing (RES) complexes from B* (Bessonov et al., 2008; Lardelli et al., 2010; Ohrt et al., 2012; Zhang et al., 2018). The resulting catalytic step I spliceosome (C complex) is remodeled by the ATPase/helicase Prp16 into a step II catalytically activated spliceosome (C* complex), in which the exon ligation reaction occurs.

In contrast to constitutive splicing, >90% of human multiexon genes undergo alternative splicing (AS) (Pan et al., 2008; Wang et al., 2008), which contributes to proteomic diversity (Keren et al., 2010; Nilsen and Graveley, 2010). Accuracy in the recognition of reactive splice sites must be compromised by flexibility in splice site choice during AS. As one of the major categories of AS, intron retention (IR) was originally thought to be nonproductive for protein production, because it often introduces a premature termination codon (PTC) into the transcript, which is subsequently targeted for degradation by nonsense-mediated decay (NMD), a cytoplasmic mRNA surveillance mechanism (Jacob and Smith, 2017; Popp and Maquat, 2013). However, Boutz et al., (2015) identified a group of intron-detaining transcripts (IDTs) in human and mouse cells that are polyadenylated, detained in the nucleus, and immune to NMD, and termed these incompletely spliced introns as detained introns (DIs). Nucleus DIs have also been documented by others and their splicing and subsequent mRNA export to the cytoplasm for protein translation has been associated with specific stimuli and stress (Ninomiya et al., 2011; Yap et al., 2012; Mauger et al., 2016; Gill et al., 2017; Naro et al., 2017; Park et al., 2017; Pendleton et al., 2017; Tan et al., 2020). Given that gene size in human is large (~27 kb in average) but RNA transcription rate is slow (~2–4 kb/min) (Tennyson et al., 1995; Lander et al., 2001; Darzacq et al., 2007; Singh and Padgett, 2009), post-transcriptional DI splicing is a quick and effective way for cells to adapt environment changes or stress. However, it remains unclear whether spliceosome is indeed paused at DIs, which catalytic step the spliceosome is paused at, what the molecular mechanisms underlie the spliceosome pausing, and what are the biological consequences of misregulation of this process.

Here, we disclose that more than one-third of cerebellum-expressed genes transcribe IDTs and ~90% of them only contain 1–2 intron(s). Using mouse forward genetics and gene knockout (KO), we demonstrate that haploinsufficiency of *Snip1* (Smad nuclear interacting protein 1) rescues IDT accumulation and neurodegeneration caused by a previously reported mutant U2 snRNA (Jia et al., 2012). SNIP1 interacts with protein components found in B^act^ spliceosome and protein components in peripheral exon junction complex (EJC), including RNPS1 (RNA binding protein with serine-rich domain 1). Like *Snip1*, knockdown of *Rnps1* rescues the DI accumulation while its overexpression is sufficient to trigger spliceosome pausing at DIs. B^act^ component and RNPS1 preferentially deposit at DIs and their surrounding sequences. Both RNPS1 docking at DIs and interaction between SNIP1 and RNPS1 are required for spliceosome pausing at DIs. *Snip1* conditional KO in cerebellum reduces DI splicing efficiency and leads to IDT accumulation and neurodegeneration. Therefore, we suggest that SNIP1 and RNPS1 function as a molecular brake to promote spliceosome pausing at highly regulated DIs, and that misregulation of this process contributes to the pathogenesis of neurodegeneration.

## Results

### A mouse forward genetic screening identifies *Snip1* as a modifier for *NMF291* phenotypes

**Figure 1.**
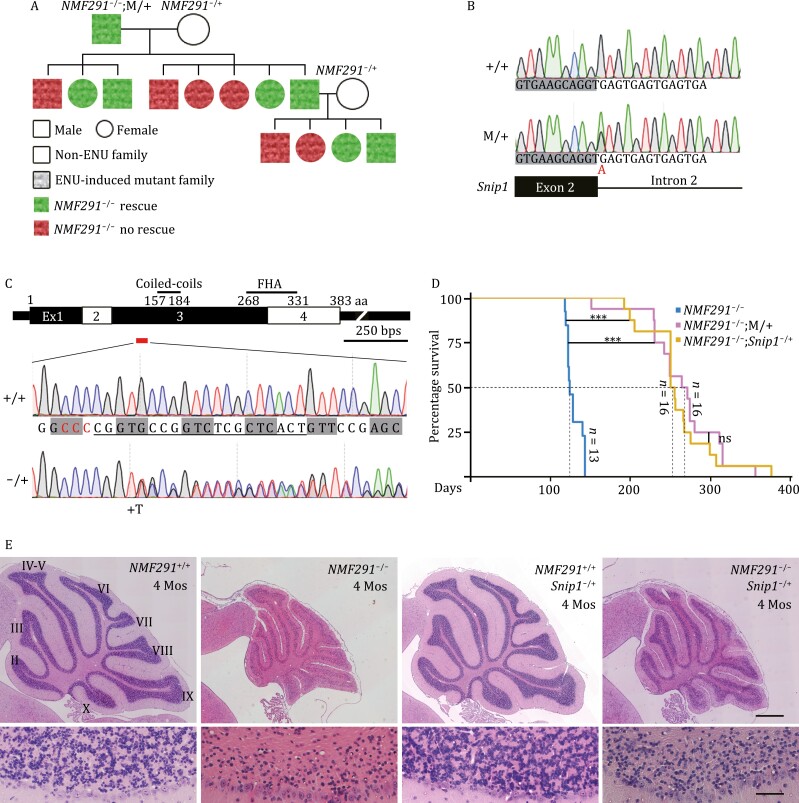
A forward genetic screening identifies *Snip1* as a modifier for *NMF291* phenotypes. (A) A *NMF291* modifier family pedigree. An ENU-induced mutant G_1_ male (*NMF291*^−/−^;M/+) carrying less ataxia phenotype (rescued, in green) was bred to an ENU-untreated *NMF291*^−/+^ (in white) female. The resulting *NMF291*^−/−^ mice were included for our phenotyping. The modifier is inherited in a dominant manner. (B) The ENU-induced modifier candidate (M/+) hits the 5ʹ SS (from GT to AT) of *Snip1* exon2 (ENSMUST00000052183.6). (C) Generation of *Snip1* KO mouse (*Snip1*^−/+^) by Crispr/Cas9. *Snip1* contains four protein-coding exons and two functional domains. Coiled-coils, 157–184 aa; FHA (forkhead-associated domain), 268–331 aa. The Cas9 editing site was labeled as a red bar. A nucleotide insertion (+T) in *Snip1* exon3 causes out-of-frame KO. PAM site was labeled in red and gRNA sequences were underlined. Codons were labeled with gray rectangles. (D) The survival curves for the indicated genotypes (***P < 0.001, ns, no statistical significance, log-rank test). Both the ENU-induced mutation (M/+) and Crispr/Cas9-generated *Snip1* KO (*Snip1*^−/+^) extended the *NMF291*^−/−^ mutant lifespans. (E) Hematoxylin and eosin-stained cerebellar sagittal sections of the indicated genotypes at 4 months (4 Mos.) of age. Lower panels, the coresponding high magnification images (lobule II). Scale bar, upper 500 μm; lower, 50 μm. See also Fig. S1, Movie 1, Table S1 and Table S2.

To understand how the previously reported mutant U2 (Jia et al., 2012) leads to global RNA splicing abnormalities and massive cerebellar granule cell loss, we established an ENU-induced mutagenesis screening for dominant modifier(s) that rescue the *NMF291*^−/−^ phenotypes (Fig. S1). A modifier (*Snip1*^M/+^) partially rescued *NMF291*^−/−^ ataxia in a dominant manner (Fig. 1A and Movie S1). One of the modifier candidates in the family was a G to A substitution that alters the 5ʹ splice site (5ʹ SS) GT of *Snip1* exon 2 to AT (Fig. 1B), which completely segregated with the rescue in the *NMF291*^−/−^ mice (Table S1). Because the ENU mutation disrupts the 5ʹ SS, we generated a *Snip1* KO mouse line by Crispr-Cas9 to examine whether *Snip1*^M/+^ rescues *NMF291*^−/−^ phenotypes through a loss-of-function mechanism (Fig. 1C). Heterozygous *Snip1* KO (*Snip1*^−/+^) rescued *NMF291*^−/−^ ataxia and significantly extended *NMF291*^−/−^ life span, comparable to the extent of *Snip1*^M/+^ (Fig. 1D). In addition, *Snip1*^−/+^ partially rescued neuron loss in the *NMF291*^−/−^ cerebellum (Fig. 1E), although *Snip1*^−/+^ itself did not show cerebellar neuron loss. We failed to harvest a homozygous *Snip1* mutant mouse for both ENU-induced mutation and Crispr-Cas9-generated KO (Table S2), indicating that *Snip1* is an essential gene and ENU-induced mutation is possibly a null allele. Therefore, we conclude that haploinsufficiency of *Snip1* rescues neurodegenerative phenotypes shown in the *NMF291*^−/−^ mutant mouse.

### Haploinsufficiency of *Snip1* globally rescues IRs in *NMF291* mutant cerebellum

**Figure 2.**
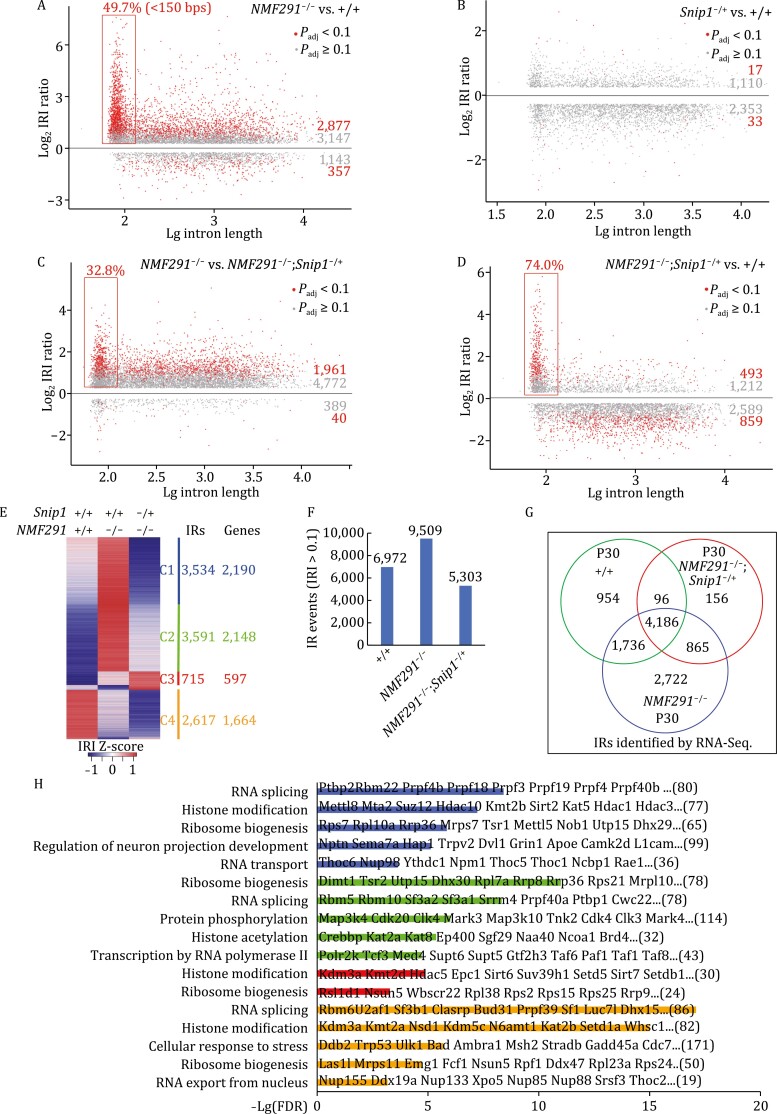
Haploinsufficiency of *Snip1* partially rescues the *NMF291* cerebellar IRs and the majority of them also exist in wild-type cerebellum. (A–D) Pairwise comparisons of cerebellar IRI between the indicated genotypes. We set two separate cutoffs colored by red and gray, respectively. Red dots: events with FDR-adjusted *P*-value (*P*_adj_) < 0.1 and the IRI fold-change (FC) > 1.2; gray dots: *P*_adj_ ≥ 0.1 and the IRI FC > 1.2. Only events with IRI > 0.1 were included for the comparison. The retained introns less than 150 bp in length were highlighted with rectangles and their percentage of all the IRs (red rots) was labeled. Mice, male, 1 month of age, *n* = 3. (E) Heatmap represents IRI changes across the indicated genotypes. Z-score was used to normalize IRIs in each row. The numbers of IRs and their corresponding genes (Genes) are shown. (F and G) Cerebellar IRs (IRI > 0.1, F) of the indicated genotypes and ages and their overlaps (G). (H) GO analysis of gene clusters shown in (E). The values in *X* axis are −*L*g adjusted *P*-values. Gene numbers in each term were labeled. Some of gene names were labeled in the plot. See also Fig. S2.

One of pathological features in *NMF291* mutant cerebellum is severe IRs (Jia et al., 2012). To quantitatively and globally interrogate whether *Snip1*^−/+^ is able to rescue the IRs, we performed cerebellar RNA-seq with poly(A) selection in wild type (+/+), *Snip1*^−/+^, *NMF291*^−/−^, and *NMF291*^−/−^;*Snip1*^−/+^ mice at 1 month of age, when the expression of mutant U2 snRNAs starts to be upregulated and IRs become severe (Jia et al., 2012). We employed intron retention index (IRI) to represent the IR levels, which were calculated by the ratio of intronic reads normalized to flanking exonic reads (Jia et al., 2012; Wong et al., 2013; Braunschweig et al., 2014). Consistent with a previous report (Jia et al., 2012), the IRI ratio of *NMF291*^−/−^ to +/+ showed more IRs in the *NMF291*^−/−^ cerebella (Figs. 2A and S2A–C), regardless of whether considering high (FC > 1.2 and *P*_adj_ < 0.1) or low (FC > 1.2 and *P*_adj_ ≥ 0.1) confidence IR events. As reported before (Jia et al., 2012), about half of high confidence IRs were small introns (intron length <150 bp). The comparative IRI ratio of *Snip1*^−/+^ to +/+ showed a slight depletion of IRs in *Snip1*^−/+^ cerebella (Fig. 2B). Pairwise comparison between *NMF291*^−/−^ and *NMF291*^−/−^;*Snip1*^−/+^ indicated that the majority of IRs was rescued by *Snip1*^−/+^ (Figs. 2C and S2A–C). Indeed, the IRI ratio of *NMF291*^−/−^;*Snip1*^−/+^ to +/+ revealed even fewer IRs shown in *NMF291*^−/−^;*Snip1*^−/+^ cerebella than that of +/+ (Fig. 2D). Among these high confident but not rescued IRs (493 shown in Fig. 2D), 74.0% are small introns with a much higher IRI ratio (median = 8.2) than that of the rest (median = 4.5), suggesting that these IRs are insensitive to haploinsufficiency of *Snip1.*

To gain detailed insights into these rescued IRs, we choose three representatives IR events in *Nop2*, *Pias4*, and *Ptbp1*, and employed IGV (Integrative Genomics Viewer) to visualize them in wild type (+/+), *Snip1*^−/+^, *NMF291*^−/−^, and *NMF291*^−/−^;*Snip1*^−/+^ cerebella. Indeed, *Snip1*^−/+^ largely rescued these IRs (Fig. S2C and S2D). In addition, we noticed higher expression levels of the corresponding genes in *NMF291*^−/−^ cerebella compared to that of other genotypes, suggesting that the higher gene expression was also largely rescued by *Snip1*^−/+^. To examine it globally, we compared expression level of the corresponding genes, whose IRs were rescued with high confidence (1961 events shown in Fig. 2C), in +/+, *NMF291*^−/−^, and *NMF291*^−/−^;*Snip1*^−/+^ cerebella (Fig. S2E). Gene expression levels comparing wild type and *NMF291*^−/−^ were mutually exclusive, with upregulated genes in the *NMF291*^−/−^ cerebellum globally rescued by *Snip1*^−/+^, and little effect on downregulated genes.

**Figure 3.**
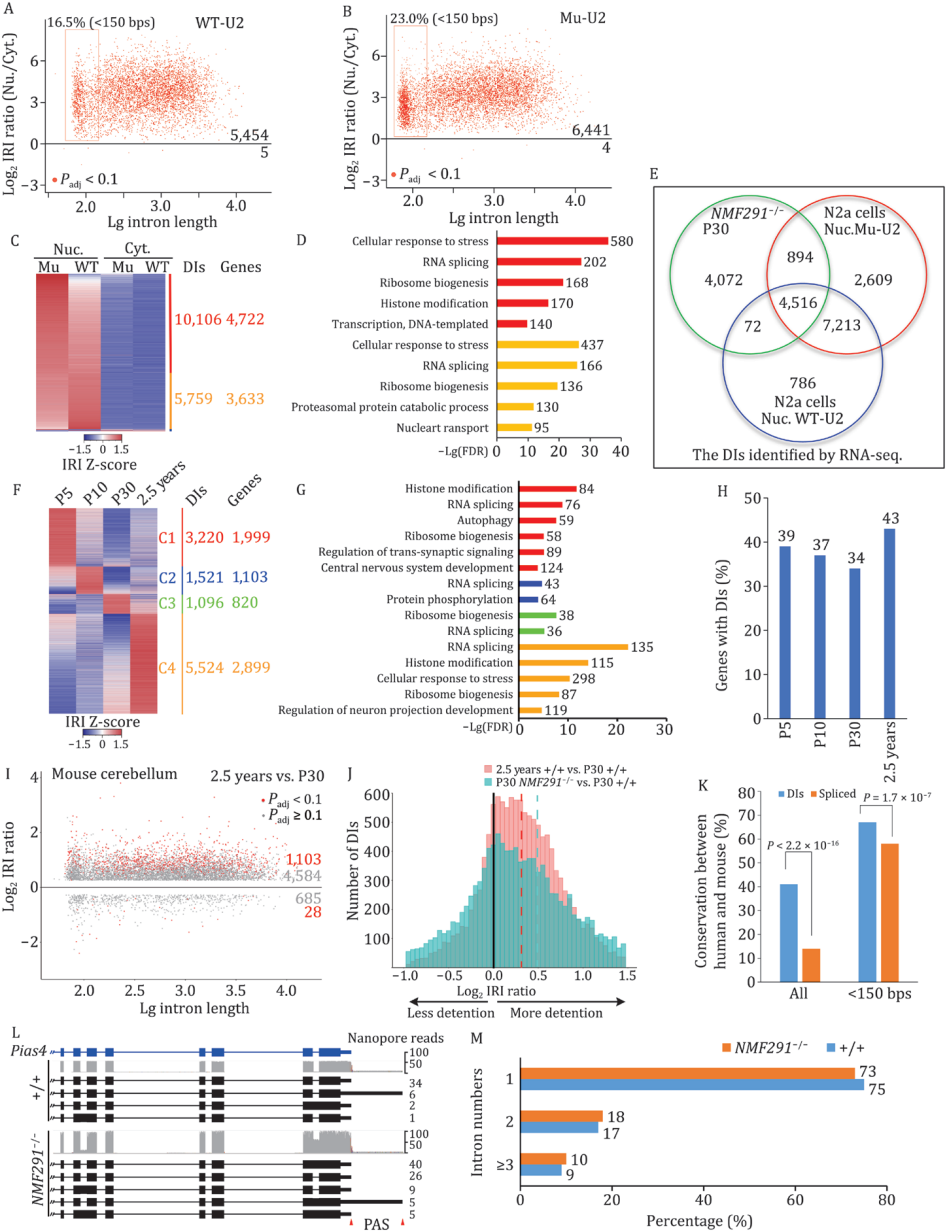
Intron-containing transcripts overrepresented in the *NMF291* mutant cerebellum are IDTs that are developmentally regulated. (A and B) The nuclear and cytosolic fractions of N2a cells expressing WT-U2 or Mu-U2 were applied for RNA-seq, respectively. The IRI ratio of nucleus to cytosol was plotted (*n* = 3, *P*_adj_ < 0.1). (C and D) Heatmap represents the DIs in nucleus (Nuc.) and cytosol (Cyt.) of N2a cells expressing WT-U2 or Mu-U2. The numbers of the DIs and their corresponding genes (Genes) are shown. GO analysis (D). The *X* axis shows –*L*g-adjusted *P*-values. Gene number in each term was labeled. (E) A majority of intron-containing transcripts overrepresented in *NMF291* mutant cerebellum also appeared in nucleus extraction of N2a cells. (F and G) Heatmap represents IRI changes across different ages by RNA-seq. We included the DIs with IRI > 0.1 and clustered them. GO analysis (G). Mice, +/+, three males for each age point. (H) Percentage of cerebellum-expressed genes with DIs detected by RNA-seq. Wildtype P5, 10, 30, and 2.5-year mouse cerebella were applied for RNA-seq. Genes with the DI surrounding exon reads >20 were considered as cerebellum-expressed genes. (I) Comparisons of cerebellar IRI between aged (2.5 years) and young (P30) mice. We set two separate cutoffs colored by red and gray as shown in Fig. 2. Mice, male, *n* = 3. (J) Distribution of DIs with their IRI changes in aged (2.5 years) and P30 *NMF291*^−/−^ mouse cerebellum compared to P30 wild-type control. *X*-axis is log_2_ adjusted IRI ratio. Dashed lines indicate medians of log_2_ adjusted IRI ratio in these two groups. (K) Evolutionary conservation of the DIs (IRI > 0.1) and efficientlyspliced (IRI < 0.02) introns detected in the *NMF291*^−/−^ mutant cerebellum by RNA-seq. *P* values correspond to two-sided proportion tests. (L) The representative DIs detected by nanopore-sequencing. Cerebellar mRNAs extracted from wild-type (+/+) and *NMF291*^−/−^ animals (1 month of age, *n* = 3). PAS, polyadenylation site. (M) A majority of DIs detected by nanopore-sequencing contain 1–2 intron(s) in wild-type (90%) and mutant (91%) cerebella. See also Figs. S3–5.

To further understand the corresponding gene functions, we extracted IRs (IRI > 0.1) from the +/+ (6972), *NMF291*^−/−^ (9509), and *NMF291*^−/−^;*Snip1*^−/+^ (5303) cerebella and compared the IR-level changes across different genotypes (Fig. 2E and 2F). Interestingly, 62.3% and 53.1% of IRs shown in the *NMF291*^−/−^ cerebella also appear in +/+ and *NMF291*^−/−^;*Snip1*^−/+^, respectively (Fig. 2G). IR events were grouped into four clusters across the different genotypes and the corresponding genes in each cluster are related to several cellular processes (Fig. 2E and 2H). For cluster 1 (C1) genes, the IRs were fully rescued by *Snip1*^−/+^ (Fig. 3A) and the corresponding genes are highly enriched in RNA splicing, histone modification, ribosome biogenesis, neuronal projection development, and RNA transport (Fig. 2H). The IRs of cluster 2 (C2) genes were less rescued by *Snip1*^−/+^. This group of genes are involved in ribosome biogenesis, RNA splicing, protein phosphorylation, histone modification, and transcription by RNA polymerase II. Cluster 3 (C3) genes are unique, and their IRs were not rescued but even overrepresented in *NMF291*^−/−^;*Snip1*^−/+^ cerebella, although their number is less than that of other clusters. These genes are enriched in histone modification and ribosome biogenesis. The IR events in cluster 4 (C4) genes were under-represented in the *NMF291*^−/−^ cerebella compared to that of +/+, and became even less-represented in *NMF291*^−/−^;*Snip1*^−/+^. These genes are enriched in RNA splicing, histone modification, and cellular response to stress. Taken together, we demonstrate that: (i) haploinsufficiency of *Snip1* globally rescues the IRs and their corresponding gene expression shown in the *NMF291* mutant cerebellum; (ii) the cerebellar IRs are not randomly distributed in their transcripts; (iii) the majority of IRs overrepresented in the *NMF291*^−/−^ cerebella also exist in that of the wildtype; and (iv) genes involved in RNA metabolism/processing and cellular response to stress tend to transcribe intron-containing transcripts.

### Intron-containing transcripts overrepresented in mutant U2 cerebellum are IDTs featured by nuclear-localized, incompletely spliced, and polyadenylated

Intron-containing transcripts often contain PTCs, triggering NMD for degradation (Maquat, 2004; Jacob and Smith, 2017). However, in *NMF291* mutant cerebellum, intron-containing transcripts are abundant and stable (Jia et al., 2012) (Figs. 2 and S2), which prompted us to examine whether these transcripts are targeted by NMD. To this end, we cultured Neuro2a (N2a) cells, a mouse neuroblastoma cell line, and transfected the cells with wild-type (WT-U2) and mutant U2 (Mu-U2) snRNA expressing plasmids. Compared to WT-U2, expression of Mu-U2 increased IRs at several endogenous sites, including *Ptbp1* intron5 (Fig. S3A). The IRs were not sensitive to inhibition of NMD, either by addition of cycloheximide (CHX) in the culture medium or by knockdown of *Upf1* (Fig. S3B–E), suggesting that they are stable and detained in nucleus (Boutz et al., 2015; Jacob and Smith, 2017). To test this possibility, we performed nuclear and cytosolic fractionation and examined IRs (Fig. S3F). Irrespective of WT- or Mu-U2 expression, IRs were enriched in the nuclear fraction. Therefore, we suggest that these incompletely spliced introns are DIs, previously featured by polyadenylated, detained in the nucleus, and immune to NMD (Boutz et al., 2015).

To globally examine the nuclear enrichment of IDTs, we performed RNA-seq with poly(A) selection in isolated nuclear and cytosolic fractions in N2a cells (Fig. 3A and 3B). The IRI ratio of the nucleus to the cytosol indicated a global nuclear enrichment of polyadenylated IDTs in these cells regardless of whether WT-U2 or Mu-U2 was expressed. Consistent with what we observed in the *NMF291* mutant cerebellum, Mu-U2 expression in N2a cells increased the number of DIs (Fig. 3C). Genes transcribing these IDTs are functionally involved in RNA metabolism/processing and cellular response to stress (Fig. 3D), similar to what we observed *in vivo* (Fig. 2H). In fact, a majority of the DIs (56.6%) in the *NMF291* mutant cerebellum also appear in N2a cells expressing Mu-U2 (Fig. 3E).

Overrepresented IDTs in *NMF29*1 mutant cerebellum (Figs. 2 and S2) allowed us to examine whether these polyadenylated IDTs produce protein or not with high confidence. *Ptbp1* transcripts with intron 5 were a minor form in the wild-type cerebellum but became dominant in the mutant (Figs. S2C and S4A). Using a PTBP1 N-terminal antibody, we detected comparable amounts of PTBP1 (~50 kDa) in wild-type and mutant cerebella but failed to detect the corresponding truncated protein supposedly produced from *Ptbp1* intron 5-containing transcripts (Fig. S4B and S4C), supporting the idea that the IDTs are detained in nucleus with limited accessibility to cytoplasmic protein translation machinery. To globally examine the protein products derived from DIs, we generated a customized peptide database, including peptides encoded by the DIs found in wild-type and *NMF291* mutant cerebellum and their upstream exons (Fig. S4D). Although we retrieved thousands of peptides coded by the upstream exons, we failed to retrieve any peptide encoded by the DIs from three biological replicates of the wild-type and *NMF291* mutant cerebellar protein lysates (Fig. S4E). Therefore, we suggest that the majority of intron-containing transcripts overrepresented by expression of mutant U2 are IDTs featured by nuclear-localized, incompletely spliced, and polyadenylated.

### More than one-third of cerebellum-expressed genes transcribe IDTs that are highly regulated and ~90% of these IDTs only contain 1–2 intron(s)

To test whether DIs are regulated during cerebellum development and aging, we analyzed cerebellar DIs (IRI > 0.1) at P5, 10, 30, and 2.5-years by using RNA-Seq and grouped them into four clusters (Fig. 3F). Every developmental age point has their unique IDTs transcribed by genes involved in several cellular processes, including RNA splicing, cellular response to stress, histone modification, and ribosome biogenesis (Fig. 3G). To examine how much percentage of cerebellum-expressed genes have DIs, we extracted 8917 genes with reasonable expression (the DI surrounding exon reads > 20) in P5, 10, 30, and 2.5-years cerebella (Fig. 3H). Among them, 34.2%–43.2% have reliable DIs (IRI > 0.1) with higher percentages in P5 and 2.5-year cerebella. The IRI ratio of 2.5-years to P30 wild type indicated an enrichment of IDTs in aged cerebellum (Fig. 3I). However, compared to the DIs overrepresented in mutant cerebellum, less detention appears in aged cerebellum, with smaller mean value of log_2_ IRI ratio (0.32 for aged, 0.48 for *NMF291*^−/−^ cerebellum, both compared to that of P30 +/+) (Fig. 3J). To understand the details of DIs at single transcript level, we employed nanopore sequencing, a long-read sequencing technology (Venkatesan and Bashir, 2011), to detect the DI features in P30 and 2.5-year wild-type and *NMF291*^−/−^ cerebella. To call for full-length IDTs, we only included the nanopore reads containing both 5ʹUTR and 3ʹUTR with IRI > 0.05. The majority of these DIs (73%) shown in the aged cerebellum also appear in that of *NMF291* mutant cerebellum (Fig. S5A). In addition, higher percentage of IDTs of all full-length transcripts we examined showed in both aged (16.0%) and *NMF291* mutant (17.5%) cerebella (Fig. S5B), compared to that of P30 wild type. These indicate that the DIs are primarily affected during aging process, presumably when the function of spliceosome declines.

Next, we asked whether these DIs are evolutionarily conserved between mouse and human. We categorized the efficiently spliced introns (IRIs < 0.02) and the DIs (IRIs > 0.1) in the *NMF291*^−/−^ mutant cerebella. Compared to the efficiently spliced introns, the DIs are significantly more conserved between human and mouse (*P* < 2.2 × 10^−16^) (Fig. 3K). For intron length of less than 150 bp, DIs also showed significantly more conserved (*P* < 1.7 × 10^−7^) than that of efficiently spliced introns.

To further learn the DI features at full-length transcript level, we retrieved 177 002 IDTs for wild-type (+/+) and 334 999 for *NMF291* mutant (*NMF291*^−/−^) cerebella by using nanopore sequencing. Consistent with our second-generation RNA-seq results, nanopore-reads showed more IDTs in mutant cerebellum than that of wild type (Fig. 3L). However, when we categorized the IDTs in terms of their intron number distribution, most IDTs contained 1–2 intron(s) in wild-type (91%) as well as in mutant (90%) cerebella (Fig. 3M). In addition, the percentage of intron number distribution between the two genotypes is almost identical with ~70% IDTs containing only one intron in both genotypes. Taken together, our findings reveal that: (i) over one-third of cerebellum-expressed genes transcribe IDTs and their DI splicing is highly regulated during cerebellum development and aging; (ii) evolutionarily these DIs are more conserved than that of efficiently spliced; and (iii) ~90% of IDTs only contain 1–2 intron(s), indicating that DIs only comprise a small proportion of the total introns transcribed in cerebellum.

### Nuclear-localized SNIP1 binds to cerebellar polyadenylated IDTs

**Figure 4.**
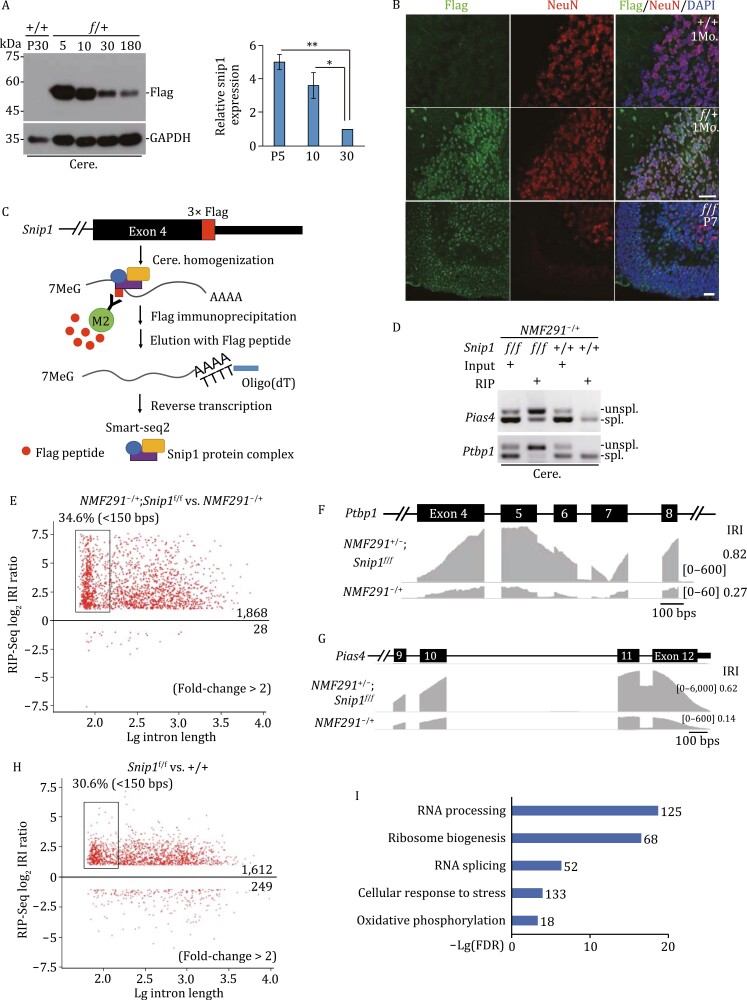
Nucleus-localized SNIP1 binds to cerebellar IDTs. (A) The expression level of SNIP1 was documented with a Flag antibody at various age points in *Snip1-Flag* KI animals. GAPDH, loading control; *f*/+, mice heterozygous for *Snip1-Flag* KI; +/+, negative control for Flag immunoblot. A summary of the expression level was inserted. (B) Flag-immunostaining was performed in *Snip1-Flag* KI mouse cerebellum at 1 month and P7 of ages. NeuN, a neuronal marker; +/+, negative control for Flag-immunostaining. Scale bar for 1 month, 30 μm; for P7, 20 μm. (C) A diagram for native Flag-RIP-seq. The RNA/protein complex containing Flag-SNIP1 was eluted by competition with free Flag peptides. The resulting Flag-SNIP1-bound RNAs were applied for Smart-seq2 to amplify full-length polyadenylated mRNAs. M2, M2-beads conjugated with Flag-antibody. (D and E) RIP was performed with cerebellar protein lysates from *NMF291*^−/+^;*f*/*f* or *NMF291*^−/+^ mice at 1 month of age. Enrichment of DIs was detected by RT-PCR in RIP products from *NMF291*^−/+^;*f*/*f* mice but not from that of *NMF291*^−/+^ controls. Global examination of the enrichment by Flag-RIP-seq from three biological replicates (E). (F and G) Two representative SNIP1-bound IDTs visualized by IGV. (H and I) Global examination of enrichment of SNIP1-bound DIs in P7 *Snip1*^*f*/*f*^ cerebellum by Flag-RIP-seq (*n* = 3). Wild-type (+/+) animals served as negative control for Flag-RIP-seq. GO analysis of the genes transcribing SNIP1-bound IDTs shown in (I). In A, the values are presented as mean ± SEM (mice, *n* ≥ 3 for each age point). *P < 0.05, **P < 0.01, ANOVA, SPSS. See also Fig. S6.

Previous studies showed that SNIP1 interacts with the TGF-β family Smad proteins, NF-κB transcription factor p65, and transcriptional coactivators p300/CBP, resulting in regulation of TGF-β and NF-κB signaling (Kim et al., 2000, 2001). Post-transcriptionally, SNIP1 also regulates Cyclin D1 RNA stability (Bracken et al., 2008). However, how SNIP1 regulates pre-mRNA splicing *per se* are not studied in mammal. If DI splicing is regulated by SNIP1, we speculated that SNIP1 must bind to these transcripts. To this end, we inserted 3× Flag tag at the C-terminal end of *Snip1* by Crispr-Cas9-mediated homologous recombination (Figs. 4A, S6A and S6B). SNIP1-Flag appeared in the knockin (KI) cerebellum at the expected molecular weight, and was absent in the wild-type control (Fig. 4A). The cerebellar expression level of SNIP1-Flag at P5 and P10 was 5.0 and 3.6 times higher than that of P30 (Fig. 4A). Ubiquitous expression of SNIP1 was documented in various adult mouse tissues (Fig. S6C).

Nuclear Flag-immunoreactive signals appeared in *Snip1-Flag* KI adult and P7 cerebella, and were absent in wild-type control (Fig. S6D). In the adult cerebellum, the majority of SNIP1-Flag signals were NeuN-positive in the internal granule layer (IGL), indicating that SNIP1 is majorly expressed in adult cerebellar granule cells. In P7 cerebellum, the Flag-immunoreactive signals were evident in proliferative granule cell progenitors in the external granule layer (EGL), migrating granule cells in the molecular layer (ML), and NeuN-positive granule cells in the IGL (Fig. 4B).

To examine whether SNIP1 binds to the post-transcriptional polyadenylated IDTs in *NMF291* mutant cerebellum, we employed a native RNA immunoprecipitation (RIP) coupled with poly(A) selection strategy (Fig. 4C). Instead of cross-linking RIP, native RIP enables us to detect entire transcripts in a more stable and long-lasting RNA-protein complex. Native Flag-RIP precipitated *Pias4* and *Ptbp1* IDTs in *NMF291* mutant cerebella expressing SNIP1-Flag (*NMF291*^−/+^;*Snip1*^*f*/*f*^), which were depleted in that of *NMF291*^−/+^ without expression of SNIP1-Flag (Fig. 4D). To globally examine the binding, we performed native RIP followed by Smart-seq2 (Picelli et al., 2013), which amplifies full-length polyadenylated mRNAs (Fig. 4C). The IRI ratio of *NMF291*^−/+^;*Snip1*^*f*/*f*^ to *NMF291*^−/+^ revealed an enrichment (1868 vs. 28) of DIs (Fig. 4E), which was also visualized in two representative *Ptbp1* and *Pias4* IDTs by IGV (Fig. 4F and 4G). Among these transcripts, 34.6% of them were small introns with a size of <150 bp (Fig. 4E).

To examine whether the binding is independent of mutant U2 expression, we performed Flag-RIP-seq in *Snip1-Flag* KI animals at P7, when SNIP1 is highly expressed (Fig. 4H). The IRI ratio of *Snip1*^*f*/*f*^ to wild type (1612 vs. 249) suggested the binding of SNIP1 to the IDTs in P7 cerebella (Fig. 4H). Among them, 30.6% are small introns with a length of <150 bp. Genes transcribing these transcripts are involved in RNA processing, ribosome biogenesis, RNA splicing, oxidative phosphorylation, and DNA repair (Fig. 4I). Therefore, we conclude that nuclear-localized SNIP1 deposits at a group of cerebellar IDTs encoded by genes involved in several cellular processes, especially RNA metabolism/processing and cellular response to stress.

### DI splicing is paused prior to the first catalytic step

**Figure 5.**
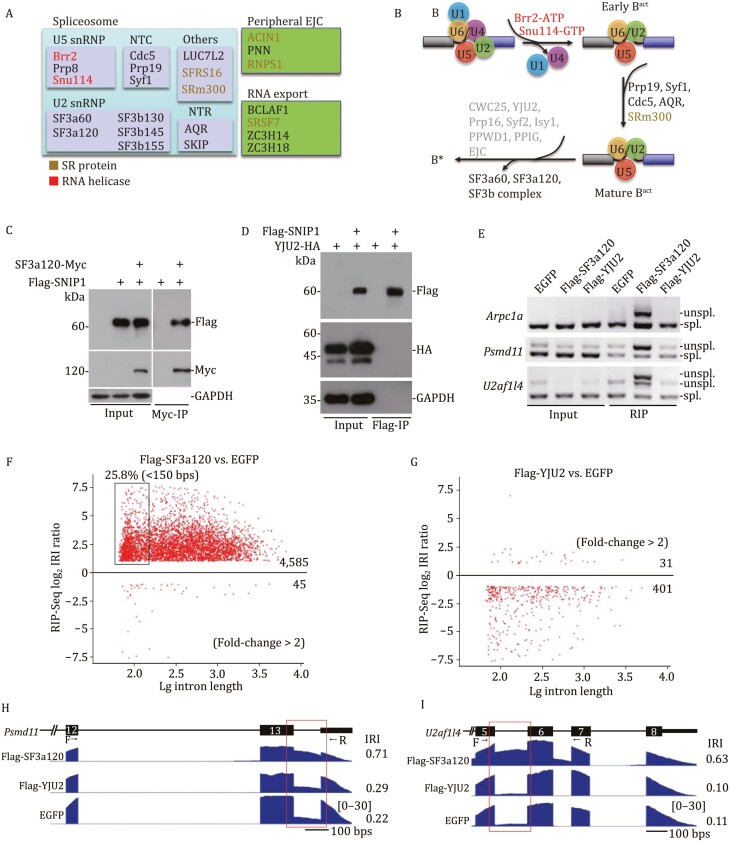
DI splicing is likely paused at B^act^. (A) Potential SNIP1 interacting proteins identified by Flag-IP coupled with mass spectrometry (co-IP/MS) in N2a cells expressing Flag-SNIP1. Many of these protein partners are involved in pre-mRNA splicing and RNA metabolism (also see Table S3). (B) The potential SNIP1 interacting partners are found in B^act^ but not B*. Factors loaded into B* are labeled in gray, which were not hit by our co-IP/MS. (C and D) Interactions between SNIP1 and protein components found in B^act^ component SF3a120 (C) but not those in B* component YJU2 (D) were validated in N2a cells expressing the indicated tagged proteins. (E) RIP was performed in N2a cells expressing Flag-SF3a120, Flag-YJU2, or EGFP. DIs were measured by RT-PCR using Smart-amplified cDNA. (F–I) Flag-RIP-seq was performed in N2a cells expressing Flag-SF3a120 (F) and Flag-YJU2 (G). N2a cells expressing EGFP, a negative control for our Flag-RIP-seq. Two representative SF3a120-bound DIs visualized by IGV (H and I). See also Table S3, Fig. S7.

To understand how SNIP1 regulates DI splicing, we examined SNIP1 protein binding partners in N2a cells, a cellular model that carries endogenous DIs (Fig. S3). In N2a cells stably expressing SNIP1-Flag, we performed Flag-co-immunoprecipitation (co-IP) followed by mass spectrometry (co-IP/MS). High confidence hits from three biological replicates were protein components found in spliceosome and peripheral EJC, or proteins involved in RNA export (Fig. 5A and Table S3). These SNIP1-interacting protein partners include RNA helicases (BRR2 and SNU114) and SR/SR-like proteins (SFRS16, SRM300, ACIN1, RNPS1, and SRSF7). We noticed that protein components found in spliceosome are that of B^act^, the activated spliceosome, but not that of B*, the catalytically activated spliceosome (Haselbach et al., 2018; Zhang et al., 2018; Wan et al., 2019) (Fig. 5B). Given that SNIP1 binds to IDTs (Fig. 4) and recent resolved human spliceosome structure also support the presence of SNIP1 in B^act^ spliceosome but not B and C complex (Bertram et al., 2017; Haselbach et al., 2018; Zhan et al., 2018; Zhang et al., 2018; Townsend et al., 2020), we proposed that DI splicing is paused at B^act^.

Release of SF3a and SF3b complexes is a key step for B^act^ to B* transition (Lardelli et al., 2010). Interactions between SNIP1 and SF3a or SF3b components, including SF3a60, SF3a120, SF3a66, and SF3b130, were validated in N2a cells expressing tagged SNIP1 (Figs. 5C and S7A–C). Although interaction between SNIP1 and Syf1, another B^act^ component, was further confirmed in the N2a cells, we failed to detect interactions between SNIP1 and B* components, including YJU2 and CWC25, in the similar conditions to those for B^act^ components (Figs. 5D, S7D and S7E). As EJC core proteins, including eIF4AIII, MAGOH, Y14, and MLN51, are recruited to the B* and C complex (Reichert et al., 2002; Bessonov et al., 2008; Zhan et al., 2018), we failed to detect the interaction between SNIP1 and eIF4AIII (Fig. S7F).

To examine whether B^act^ is indeed found at DIs, we expressed Flag-tagged SF3a120, a B^act^ component, and YJU2, a B* and C component and step-I specific factor (Haselbach et al., 2018; Zhan et al., 2018; Zhang et al., 2018; Wan et al., 2019; Townsend et al., 2020), in N2a cells and performed Flag-RIP. SF3a120 precipitated the IDTs, like *Arpc1a*, *Psmd11*, and *U2af1l4*, which were not enriched by YJU2 or EGFP controls (Fig. 5E). To globally test the association of SF3a120 with IDTs, we performed Flag-RIP-seq in N2a cells expressing Flag-SF3a120, Flag-YJU2, or EGFP. The IRI ratio of Flag-SF3a120 to EGFP showed an enrichment (4585 vs. 45) of IDTs (Fig. 5F). In contrast, the IRI ratio of Flag-YJU2 to EGFP indicated no such enrichment (31 vs. 401) (Fig. 5G). The enrichment was visualized by IGV in two representative DI events (Fig. 5H and 5I). Therefore, we suggest that DI splicing is likely paused at B^act^ prior to the first catalytic step of pre-mRNA splicing.

### SNIP1 and RNPS1 function as molecular brake to promote spliceosome pausing at DIs

**Figure 6.**
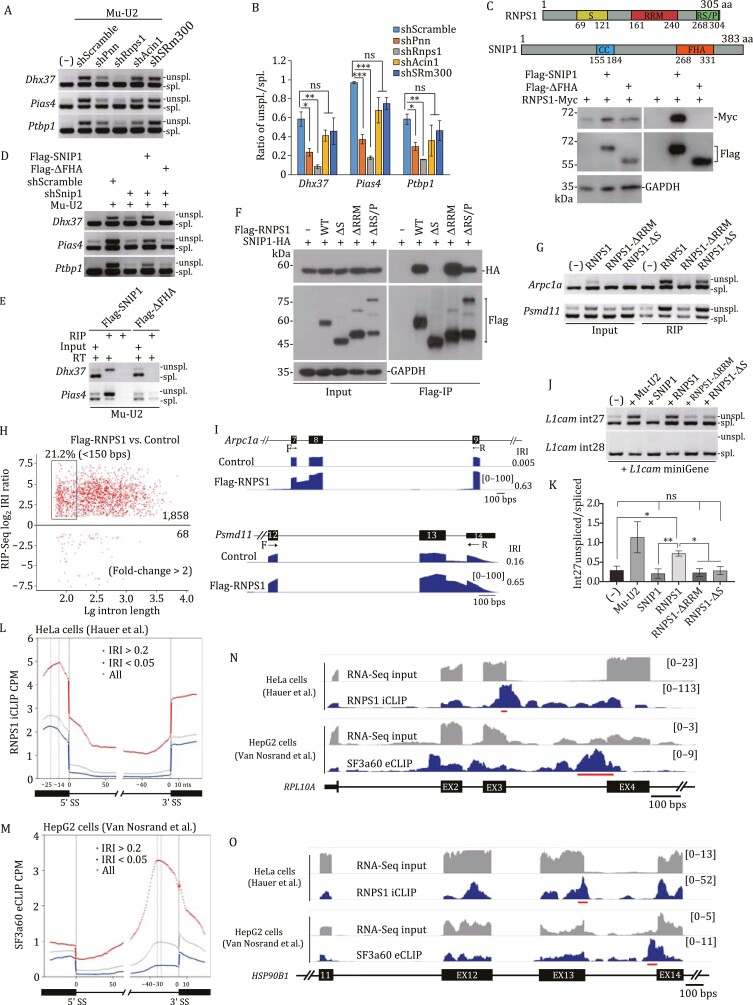
SNIP1 and RNPS1 function as a molecular brake for spliceosome pausing at DIs. (A and B) Effect of the knockdowns of genes encoding peripheral EJC components (*Acin1*, *Pnn*, and *Rnps1*) and a splicing factor (*SRm300*) on the DIs (A) amplified by expression of mutant U2 (Mu-U2) in N2a cells and the data summary (B). (C) Annotated domains of SNIP1 and RNPS1 (upper). Co-IP was performed in N2a cells expressing the Flag-tagged full-length or FHA domain-truncated SNIP1 (ΔFHA) together with RNPS1-Myc (lower). (D) N2a cells were infected with scrambled shRNA or shSnip1 and transfected with the indicated expression plasmids. (E) Flag-RIP was performed with protein lysates from N2a cells infected with Flag-tagged full-length or ΔFHA SNIP1 and transfected with Mu-U2 expression plasmid. RT, reverse transcriptase. (F) Co-IP was performed in HEK293 cells expressing HA-SNIP1 and/or Flag-tagged RNPS1, including full length and various domain-truncated RNPS1. (G) Flag-RIP was performed with protein lysates from N2a cells expressing the indicated proteins. Smart-amplified cDNA was analyzed by RT-PCR. (H and I) Global examination of the RNPS1-bound DIs in N2a cells expressing Flag-RNPS1. Control, naïve N2a cells. Two representative RNPS1-bound DIs visualized by IGV (I). (J and K) HEK293 cells were transfected with previously described splicing minigene reporters derived from *L1cam* detained intron 27 (int27) and constitutively spliced intron 28 (int28) (PMID: 22265417). The data summary was shown in (K). (L and M) CLIP-seq read density plot for RNPS1 (L) and SF3a60 (M). Read density plot with DIs (IRI > 0.2) shown in red; read density plot with efficientlyspliced introns (IRI < 0.05) shown in blue; read density plot regardless of IRI shown in gray. RNPS1 iCLIP data from ArrayExpress (PMID: 27475226) and SF3a60 eCLIP data from ENCODE (PMID: 27018577). (N and O) Read density tracks along two representative genes *RPL10A* (N) and *HSP90B1* (O) viewed by IGV and scaled by RPM (reads per million usable). The most significant clusters called by CLIPper and illustrated by the red lines. In (A, D, E, G, and J), DIs were measured by RT-PCR. Unspl., unspliced transcripts; spl., spliced transcripts. In (B and K), the values are presented as mean ± SEM, *n* = 3. *P < 0.05, **P < 0.01, ***P < 0.001, ns, no statistical significance, ANOVA, SPSS. See also Fig. S8.

As we documented above, haploinsufficiency of *Snip1* partially rescues DIs from accumulating in *NMF291* mutant cerebellum (Fig. 2). In N2a cells, knockdown of *Snip1* by shRNA (sh-Snip1) also reduced the levels of several endogenous intron detention events that were amplified by the expression of Mu-U2 (Fig. S8A and S8B). In addition to protein components found in B^act^, protein components of the peripheral EJC (ACIN1, PNN, and RNPS1) were also identified as potential SNIP1 interacting partners (Table S3). Like *Snip1*, knockdown of *Pnn* and *Rnps1* significantly reduced the intron detention amplified by Mu-U2 (Figs. 6A, 6B and S8C). Interaction of SNIP1, PNN, and RNPS1 was confirmed by co-IP in N2a cells simultaneously expressing tagged PNN, SNIP1, and RNPS1 (Fig. S8D), suggesting that these proteins work together to regulate spliceosome pausing. Although splicing factor SRm300 was found by our co-IP/MS (Table S3), SRm300 knockdown did not significantly influence the intron detention we examined (Fig. 6A and 6B).

Interaction between SNIP1 and RNPS1 depends on SNIP1 FHA (forkhead-associated) domain, a small protein module involved in phospho-dependent protein/protein interaction (Durocher and Jackson, 2002; Wysoczanski et al., 2014), because deletion of the FHA domain (Flag-ΔFHA) abolished the SNIP1-RNPS1 interaction (Fig. 6C). Exogenously expressed full-length SNIP1 but not ΔFHA SNIP1 restored intron detention amplified by Mu-U2 in N2a cells infected with shSnip1, suggesting that interaction between SNIP1 and RNPS1 is required for spliceosome pausing at DIs (Figs. 6D and S8E). In addition, unlike Flag-SNIP1, Flag-ΔFHA failed to precipitate IDTs (Fig. 6E), suggesting that SNIP1 binds to the IDTs through interaction with RNPS1.

To examine how RNPS1 interacts with SNIP1, we expressed full-length and various truncated forms of RNPS1, including ΔS (a serine-rich domain deletion), ΔRRM (an RNA recognition motif deletion), and ΔRS/P (an arginine and serine/proline-rich domain deletion), together with SNIP1 in N2a cells (Fig. 6F). ΔS abolished RNPS1-SNIP1 interaction but ΔRRM and ΔRS/P did not, suggesting that RNPS1 interacts with SNIP1 through its serine-rich domain.

Unlike RNPS1, SNIP1 does not contain an annotated RNA binding domain. The RRM of RNPS1 is involved in formation of peripheral EJC complexes, which in turn facilitate RNA binding (Murachelli et al., 2012; Boehm et al., 2018). Flag-RNPS1 precipitated IDTs in N2a cells expressing Mu-U2, and this was absent in control cells not expressing the Flag-RNPS1 (Fig. S8F). In addition, RNPS1 binds to IDTs independent of Mu-U2 expression (Fig. 6G). However, the binding was abolished by RNPS1-ΔRRM but not RNPS1-ΔS, suggesting that RRM but not interaction between SNIP1 and RNPS1 is required for the binding. Our Flag-RIP-seq data further support the idea that RNPS1 binds to IDTs (Fig. 6H and 6I). Among the RNPS1-bound DIs, 21.2% of them are small introns with a length of <150 bp (Fig. 6H).

If interaction between SNIP1 and RNPS1 is required for spliceosome pausing at highly regulated DIs, we assumed that ΔS RNPS1 would lose its ability to modulate the pausing. To this end, we employed two previously reported splicing reporters derived from two neighboring *L1cam* introns, one detained intron 27 (int27) and one constitutively spliced intron 28 (int28) (Jia et al., 2012). Severe intron detention of int27 shown in the *NMF291* mutant cerebellum was rescued by *Snip1*^−/+^ (Fig. S2B), suggesting that SNIP1- and RNPS1-containing complex modulates spliceosome pausing at int27. As previously reported (Jia et al., 2012), expression of Mu-U2 decreased the splicing efficiency of int27 but did not affect that of int28 constitutive splicing (Fig. 6J and 6K). Expression of full-length RNPS1, but not SNIP1, significantly decreased int27 splicing but did not affect int28 splicing, suggesting that RNPS1 is sufficient to induce intron detention. However, both ΔS and ΔRRM abolished RNPS1-mediated intron detention. Therefore, we suggest that RNPS1 docks at DIs through its RRM to induce RNPS1–SNIP1 interaction, which in turn functions as molecular brake to pause spliceosome at highly regulated DIs.

### RNPS1 and SF3a60 dock at different positions of DIs

EJC core proteins and peripheral EJC component RNPS1 have been documented to bind to mRNA and involved in post-transcriptional mRNA processing (Hayashi et al., 2014; Malone et al., 2014; Le Hir et al., 2016; Blazquez et al., 2018; Boehm et al., 2018; Gonatopoulos-Pournatzis et al., 2018). To examine the binding sites of RNPS1 and SF3a complex at DIs, we reanalyzed previous reported RNPS1- and SF3a60- cross-linking and immunoprecipitation (CLIP) data (Hauer et al., 2016; Van Nostrand et al., 2020a, b). Globally, RNPS1 CLIP-seq reads piled up at 5ʹ-exons (with a binding peak at −14 nucleotides (nts) of 5ʹ SS) and 5ʹ-introns (between 0 and 30 nts of 5ʹ SS) of the DIs (IRI > 0.2) (Fig. 6L), slightly differing from the binding site of core EJC (−24 to −20 nts from the 5ʹ SS) (Hauer et al., 2016; Le Hir et al., 2016). For SF3a60—a protein component of the SF3a complex—the CLIP-seq reads concentrated between −40 and 0 nts of the 3ʹ SS of DIs, with a peak at −30 nts (Fig. 6M). However, such RNPS1- and SF3a60-CLIP-seq peaks were less represented at the positions of the efficientlyspliced introns (IRI < 0.05) or all the introns we examined (All).

For representative CLIP peaks at DIs, IGV illustrated that intron 4 of *RPL10A* and intron 13 of *HSP90B1* are the highest confidence DIs compared to neighboring introns in both Hela and HepG2 cells (Fig. 6N and 6O). The most stringent RNPS1 binding peaks identified by CLIPper appeared at the 5ʹ exon/intron boundary of the highest confidence DIs. The SF3a60 peaks were located inside of the highest confidence DIs close to the 3ʹ SS. Taken together, our analysis suggests that RNPS1, a peripheral EJC component, and SF3a60, an SF3a complex component, preferentially dock at DIs.

### Conditional KO of *Snip1* in cerebellar granule cells leads to IDT accumulation and neurodegeneration

**Figure 7.**
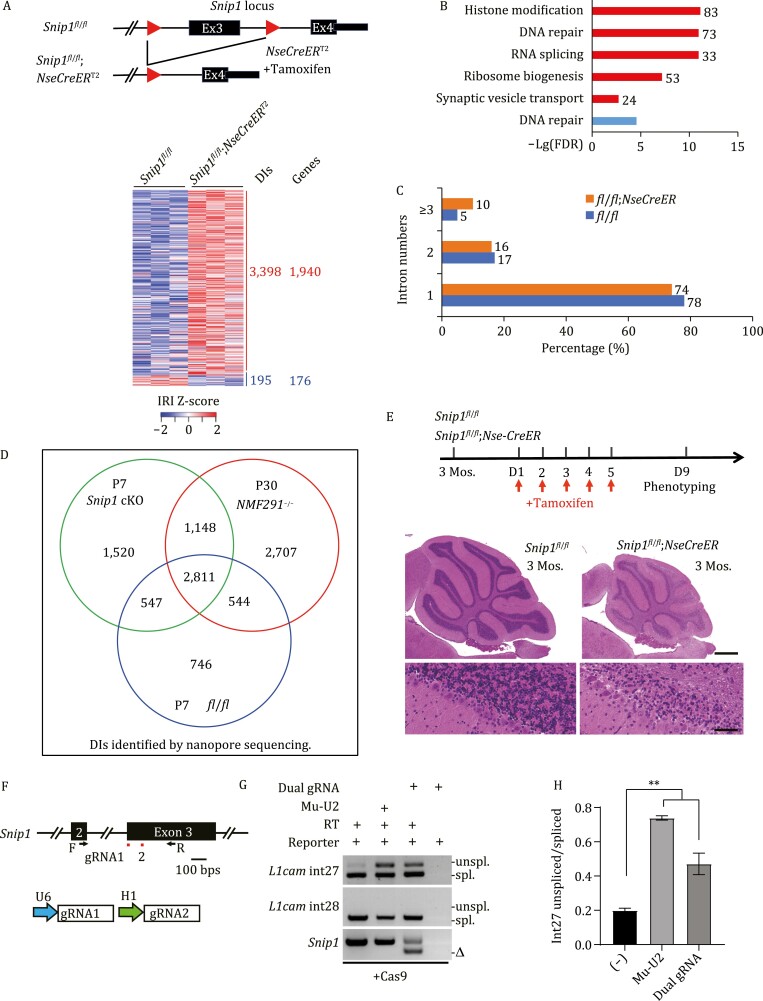
*Snip1* cKO in cerebellar granule cells leads to IDT accumulation and neurodegeneration. (A) A schematic diagram for Cre-dependent *Snip1* cKO (upper). Cerebellar RNAs extracted from the indicated genotypes on P7 (*n* = 3) were applied for RNA-seq. Events with IRI > 0.1 were included for clustering. (B) GO analysis of genes transcribing intron-containing transcripts that are over-represented in *Snip1* cKO cerebellum. (C) The cerebellar RNAs described in (A) were applied for nanopore sequencing. Intron number distribution of the indicated genotypes was plotted. (D) Intron-containing transcripts detected by nanopore sequencing in P7 *Snip1* cKO cerebellum are shared by that in P30 *NMF291*^−/−^ cerebellum. (E) Our experimental procedure: tamoxifen injections on day 1 (D1), 2, 3, 4, and 5 after 3 mos. of age; mouse phenotyping on D9 (upper). Hematoxylin and eosin-stained cerebellar sagittal sections of the indicated genotypes (lower). (F) Previously reported DECKO (Double Excision CRISPR Knockout, PMID: 26493208) approach was adopted for *Snip1* KO in N2a cells. *Snip1* dual gRNAs target the exon 3 (upper). The dual gRNAs are driven by U6 and H1 promoters, separately (lower). F  and R, primers for Crispr/Cas9-mediated editing site fusion. (G and H) N2a cells expressing *L1cam* minigenes were transfected with Mu-U2 or *Snip1* dual gRNAs plasmids. Cas9 was constitutively expressed in these cells. DIs were measured by RT-PCR. Unspl., unspliced transcripts; spl., spliced transcripts. Δ, Crispr/Cas9-mediated editing site fusion at RNA level measured by RT-PCR. Primers used for fusion detection were labeled in (F). The data summary shown in (H). In E, the corresponding high magnification images from lobule II. Scale bar, upper 200 μm; lower, 100 μm. In (H), data are presented as mean ± SEM, **P < 0.01, *n* = 4, one-way ANOVA, SPSS. See also Figs. S9 and S10).

SNIP1 is expressed in granule cells in the developing and adult cerebellum (Fig. 4A and 4B) and homozygous *Snip1* KO leads to embryonic lethality (Table S2). To reveal the biological consequences of *Snip1* KO in the cerebellum, we generated a *Snip1* floxed mouse line and crossed it to *Nse-CreER*^*T2*^, a tamoxifen-inducible Cre line specific for Cre expression mainly in cerebellar granule cells and a few granule neurons in the hippocampal dentate gyrus (Fig. S9A and S9B) (Pohlkamp et al., 2014). We confirmed the specificity of this system by crossing the *Nse-CreER*^*T2*^ to a Cre-dependent reporter line, *H2B mCherry* (Peron et al., 2015) (Fig. S9C). After tamoxifen injections on P3, P4, and P5, we observed a deletion of *Snip1* exon 3 at both the genomic DNA and mRNA levels, specifically in the cerebellum and hippocampus but not in the cortex of *Snip1*^*fl*/*fl*^;*NseCreER*^*T2*^ mice on P9 (Fig. S9D). Such deletions did not appear in the brain regions in age-matched *Snip1*^*fl*/*fl*^ controls. We harvested P7 cerebella from *Snip1*^*fl*/*fl*^;*NseCreER*^*T2*^ and age-matched *Snip1*^*fl*/*fl*^ mice after tamoxifen injections and performed RNA-seq (Fig. 7A). Accumulation of intron-containing transcripts was evident in the *Snip1* conditional KO (cKO) cerebella compared to controls. Genes transcribing these transcripts are functionally involved in histone modification, DNA repair, RNA splicing, ribosome biogenesis, and synaptic vesicle transport (Fig. 7B), similar to what we observed in the *NMF29*1 mutant cerebellum (Fig. 2H). To understand the details of incompletely spliced introns induced by *Snip1* cKO at the single transcript level, we sent the cKO cerebella for nanopore sequencing. Similar to what we observed in wild-type and *NMF291* mutant cerebella (Fig. 3K and 3L), most intron-containing transcripts had one or two intron(s) in both *Snip1* cKO (for one, 74%; for two, 16%) and an age-matched control (*Snip1*^*fl*/*fl*^, for one, 78%; for two, 17%) (Fig. 7C). These data suggest that like the *NMF291* mutation, *Snip1* cKO in cerebellar granule cells leads to accumulation of IDTs, but has less effect on constitutive splicing. Indeed, the majority of intron-containing transcripts (65.7%, 3959/6026) present in *Snip1* cKO cerebella on P7 are also found in *NMF291* cerebella on P30 (Fig. 7D).

As IDT accumulation and neurodegeneration evidenced in the *NMF291* mutant cerebellum (Jia et al., 2012) and the IDTs largely overlapped between *Snip1* cKO and *NMF291*^−/−^ cerebella (Fig. 7D), we then asked whether *Snip1* cKO in adult cerebellum also leads to neurodegeneration. To this end, we injected tamoxifen into adult *Snip1*^*fl*/*fl*^;*NseCreER*^*T2*^ animals and age-matched controls (Fig. 7E). Nine days after injection, we observed massive granule cell loss in all cerebellar lobules. To examine whether *Snip1* KO like the *NMF291* mutation impairs splicing efficiency of DI, we employed the *L1cam* splicing reporters (Jia et al., 2012). In order to achieve *Snip1* KO in N2a cells, we employed dual-gRNA approach (Aparicio-Prat et al., 2015) and the high fusion rate of two gRNA cutting sites at RNA level was evidenced by RT-PCR (Fig. 7F and 7G). Both Mu-U2 expression and *Snip1* KO in N2a cells significantly reduced splicing efficiency of int27 but had little effect on that of constitutive splicing of int28 (Fig. 7G and 7H). Taken together, we demonstrate that *Snip1* cKO in cerebellar granule cells cell-autonomously leads to neurodegeneration and *Snip1* KO impairs splicing efficiency at highly regulated DIs but not constitutively spliced introns.

## Discussion

Here, we describe interaction between SNIP1 and RNPS1 as a molecular brake to promote spliceosome pausing at highly regulated DIs. Together with a previous report (Jia et al., 2012), we suggest that misregulation of this process contributes to pathogenesis of neurodegeneration.

The yeast homolog of SNIP1 is Pml1 (pre-mRNA leakage protein 1). Compared to human SNIP1 (396 aa), yeast Pml1 (204 aa) lacks 1–180 aa N terminus and has as low as ~30% similarity to human SNIP1. Yeast RES complex contains Snu17, Bud13, and Pml1, corresponding to human RBMX2, BUD13, and SNIP1 (Dziembowski et al., 2004). RES complex is initially recruited to the B/B^act^ complex and released from B* (Fabrizio et al., 2009; Ohrt et al., 2012; Wysoczanski et al., 2014; Schneider et al., 2015; Yan et al., 2016; Zhang et al., 2018; Wan et al., 2019). We demonstrate that SNIP1 interacts with protein components found in B^act^ but not in B* (Fig. 5A–D). In yeast, unlike spliceosome basal components, such as SF3b subunits, the RES complex is not essential for yeast growth (Gottschalk et al., 2001; Dziembowski et al., 2004). In zebrafish, individual KO of *bud13*, *snip1*, and *rbmx2* leads to mis-splicing a subset of introns (Fernandez et al., 2018). In mammalian cells, BUD13 binds to a group of retained introns and regulates their splicing (Frankiw et al., 2019). It is plausible that SNIP1 functions together with the other two RES complex proteins to regulate spliceosome pausing at DIs, although our SNIP1 co-IP/MS experiments did not achieve reliable RBMX2 and BUD13 hits (Table S3). Given that histone modification plays an important role in alternative splicing regulation and SNIP1 regulates p300 histone acetyltransferase activity (Kim et al., 2000, 2001; Luco et al., 2010; Xing et al., 2014), SNIP1 could also indirectly regulate DI splicing.

Previous studies demonstrated that RNPS1 interacts with NMD machinery for mRNA surveillance (Le Hir et al., 2000; Lykke-Andersen et al., 2001; Gehring et al., 2005; Hauer et al., 2016). RNPS1 interacts with SAP18, ACIN1, or PNN to form the heterotrimeric apoptosis and splicing-associated protein (ASAP) complex (ACIN1, SAP18, and RNPS1) or the PSAP complex (PNN, SAP18, and RNPS1) (Schwerk et al., 2003; Tange et al., 2005; Murachelli et al., 2012). Both ACIN1 and PNN contain a similar RNPS1-SAP18-binding (RSB) motif to interact with RNPS1 and SAP18, suggesting that the formation of the ASAP and PSAP complexes is mutually exclusive and the resulting complexes determine the specificity of RNA substrate binding (Murachelli et al., 2012). This may explain why knockdown of *Rnps1* or *Pnn* but not *Acin1* significantly reduced the intron detentions induced by the expression of mutant U2 (Fig. 6A and 6B). Our experiments did not support that SNIP1 reliably interact with the core EJC components (Figs. 5A and S6F). Given that ACIN1 and PNN are located in nuclear speckles—where post-transcriptional splicing occurs—it is plausible that RNPS1 regulates post-transcriptional splicing by temporarily forming the ASAP or PSAP complex, which is replaced by the core EJC complex during mRNA export (Mayeda et al., 1999; Li et al., 2003; Schwerk et al., 2003; Sakashita et al., 2004; Girard et al., 2012).

RNPS1 docking at DIs is sufficient to induce intron detention but has little effect on constitutive splicing (Fig. 6J and 6K). In addition, the interaction between SNIP1 and RNPS1 is required for spliceosome pausing at DIs (Fig. 6C–J). We propose that SNIP1 and RNPS1 function a molecular brake to promote spliceosome pausing (Fig. S10). That is why partial loss of function of SNIP1 or RNPS1, probably through releasing the molecular brake, rescues intron detentions caused by mutant U2. SNIP1 contains an FHA domain, which is involved in phospho-dependent protein–protein interaction (Durocher and Jackson, 2002; Wysoczanski et al., 2014), and the FHA domain is required for the interaction between SNIP1 and RNPS1 (Fig. 6C). Therefore, phosphorylation/dephosphorylation status of RNPS1, especially in its S domain, may participate in the spliceosome pausing/resuming at highly regulated DIs. In fact, RNPS1 phosphorylation close to its S domain was proposed to regulate splicing *in vitro* and inhibition of SR protein kinases (Clk family members) were reported to promote the post-transcriptional splicing of DIs (Trembley et al., 2005; Ninomiya et al., 2011; Boutz et al., 2015).

Like the *NMF291* mutation, *Snip1* KO decreases the splicing efficiency of DIs but has little effect on that of constitutively spliced introns (Figs. 3A–C and 7). These indicate that splicing of constitutively spliced introns and DIs has different kinetics. Most likely, constitutive introns are co-transcriptional spliced but DIs undergo post-transcriptional splicing (Girard et al., 2012) (Fig. S10). In yeast, RES complex is not required for B complex formation but for the efficient transformation from B to B^act^ (Bao et al., 2017). Here, we suggest that SNIP1 may have two sides in regulation of DI splicing: (i) to form molecular brake together with RNPS1 to pause the spliceosome at DIs; and (ii) to facilitate the spliceosome transformation at DIs. Due to different splicing kinetics, DI but not constitutive intron splicing is primarily affected in the presence of mutant (the *NMF291*^−/−^) or dysfunction (*Snip1*^−/−^) spliceosome (Fig. S10). Partial loss of SNIP1 or RNPS1 rescues mutant-U2 caused intron detentions probably through releasing the molecular brake, while complete removal of *Snip1* may impair the spliceosome transformation at DIs, thus lower the splicing efficiency (Fig. S10). Global accumulation of DIs will sequester spliceosome pausing complex, which in turn lowers the available spliceosome abundance and worsens DI splicing. Indeed, decreased amounts of core and non-core spliceosome components lead to accumulation of intron-containing transcripts (Ullrich and Guigo, 2020). In addition, accumulation of intron-containing transcripts has been documented in patient brains with neurodegenerative diseases (Adusumalli et al., 2019; Wang et al., 2020). Given that genes involved in RNA metabolism/processing and cellular response to stress tend to transcribe IDTs, global accumulation of DIs will in turn further damages these gene functions. The mechanisms underlying post-transcriptional DI splicing we describe here may help to improve the understanding of pathogenesis of neurodegenerative diseases and to develop intervention strategies in the future.

## Materials and methods

### Mice

Mice were housed in isolated ventilated cages (maximum six mice per cage), on a 12/12-h light/dark cycle, 22°C–26°C, 40%–70% humidity with sterile pellet food and water ad libitum. Cages were checked daily to ensure animal welfare. Body weight was assessed regularly to ensure no weight loss. Whenever animals were used for research, we followed the 3Rs (replacement, refinement, or reduction) rules. The C57BL/6J and ICR mice were purchased from Charles River Laboratories, Beijing, China. The Nse-CreER^T2^ line was imported from The Jackson Laboratory (JAX, Stock No. 022763) (Pohlkamp et al., 2014). For tamoxifen injection, we followed previous reports (Pitulescu et al., 2010; Lizen et al., 2015). In brief, tamoxifen base (T5648-Sigma) was dissolved in corn oil and prepared freshly before use.

### ENU mutagenesis and modifier identification

For ENU-induced mutagenesis, the *NMF291*^−/+^ males were i.p. injected with ENU (80–110 mg/kg Body Weight (B. W.)) for three consecutive weeks, as previously reported (Salinger and Justice, 2008; Chen et al., 2020). After an infertility test, the ENU-treated males were crossed to untreated *NMF291*^−/+^ females for G_1_. Of these offsprings, the *NMF291*^−/−^ mice were used for behavioral tests, and the mice with less ataxia phenotype and improved lifespan were selected for family pedigree determination.

For identification of the modifier candidates, the ENU family members with or without phenotypic improvement were applied for exome sequencing. For exome capture and library construction, the instructions of NimbleGen SeqCap EZ Exome Library SR Platform (Roche) were followed. Briefly, genomic DNA was fragmented to 200–300 bp with ultrasonic shearing. End-repair, A-tailing, adapter ligation, and pre-capture ligation were performed by using a KAPA LTP Library Preparation Kit (Roche). After exome capture, the resulting samples were amplified by KAPA HiFi HotStart Ready Mix using Pre-LM-PCR Oligos 1 and 2. Libraries passing QC were sequenced (Illumina HiSeq 2500 platform) and data processing and variant discovery were performed by using GATK platform (McKenna et al., 2010). Variant annotation and classification were achieved using ANNOVAR (Wang et al., 2010).

### Generation of *Snip1* KO, Flag-tag KI, and conditional KO mice

The CRISPR design website was used to design gRNAs and avoid off-targets (Hsu et al., 2013; Ran et al., 2013). Cas9 mRNA, gRNA (s), and/or donor DNA were injected to C57BL/6J embryos. Then the injected embryos were transferred to the oviduct ampulla of pseudo-pregnant ICR female mice. For generation of the *Snip1* KO mouse, the gRNA target sequence was AGTGAGCGAGACCGGCACCGGGG (with PAM site underlined). For generation of the *Snip1* Flag-tagged mouse, the gRNA target sequence was GGGACGGTTTCTAACAGTAGAGG. Donor DNA had two homology arms (~200 bps each) flanking the mutant PAM site. For generation of the *Snip1* floxed mouse, we employed multiple gRNAs to increase homologous recombination. The gRNAs were: gRNA-A1: GAGTCTAACTGGCCCTTCGGGGG, gRNA-A2: CAATGGTACCATCCTTTAACAGG, gRNA-B1: AGTGTGGTTCTTCCCCCGAAGGG, gRNA-B2: CTGTTAAAGGATGGTACCATTGG. Two LoxP sites were placed in the *Snip1* intron 2 and intron 3, respectively, away from conserved intronic sequences. The donor DNA contained the targeted exon 3, the flanking two LoxP sites, and two homology arms (~800 bps each). C57BL/6J mouse genomic DNA was used as a template to amplify the sequences and the homology arms in the donor DNAs. Mice with the right genotypes were further crossed to C57BL/6J mice for at least three generations to establish the lines.

### Hematoxylin and eosin staining

Mice were anesthetized and transcardially perfused with Phosphate buffered saline (PBS) and then Bouin’s solution (Sigma-Aldrich). After post-fixing in Bouin’s solution, the brain tissues were paraffin embedded. The paraffin sections were applied for hematoxylin and eosin (H&E) staining. The stained sections were scanned with Pannoramic Digital Slide Scanners (3DHISTECH).

### Cell culture, plasmid construction, transfection, lentiviral infection, shRNA knockdown, and NMD reporter assay

HEK293FT and Neuro-2a cells were cultured in Dulbecco’s modified Eagle’s medium (DMEM, Corning) complemented with 10% FBS, 1% penicillin–streptomycin in 5% CO_2_ at 37°C. For plasmid construction, genes were cloned into pCMV-3tag-4A and pCMV-3tag-1A (Agilent Technologies). In addition, we replaced EGFP and Cas9 from pLJ-EGFP and LentiCas9-Blast (Addgene) with genes of interest. Cell transfection was performed using Lipofectamine 3000 (Thermo Fisher Scientific). shRNA clones were purchased from lentiviral vector-based shRNA libraries (MISSION shRNA library, Sigma-Aldrich). Lentivirus packaging was performed as previous reported (Shalem et al., 2014). The NMD reporter assay in N2a cells was performed as previously described (Boelz et al., 2006). Briefly, N2a cells were transfected with pCI-Renilla/β-globin and pCI-firefly reporters (gifted from Drs. Gabriele Neu-Yilik and Andreas E. Kulozik). After 24 h, cells were treated with 100 μg/mL CHX or 0.01% DMSO for 5 h. Renilla and firefly luciferase were detected using Dual-Luciferase Reporter System (Promega).

### Antibodies and immunofluorescence

Antibodies used in this study were: mouse anti-FLAG (Abmart, M2008H), rabbit anti-FLAG (Sigma, F7425), mouse anti-TUBULIN (Sigma, T6793), mouse anti-HA (Abcam, ab130275), rabbit anti-HA (Invitrogen, 715500), mouse anti-Myc (Abmart, M20002), rabbit anti-NeuN (Cell Signaling, 24307), anti-c-Myc Magnetic Beads (Thermo Fisher Scientific, 88842), donkey anti-mouse IgG secondary antibody (Alexa Fluor 555, Thermo Fisher Scientific, A31570), donkey anti-rabbit IgG secondary antibody (Alexa Fluor 555, Thermo Fisher Scientific, A31572), donkey anti-goat IgG secondary antibody (Alexa Fluor 488, Thermo Fisher Scientific, A11055). PTBP1 N-terminal antibody (PTB-NT, generated from 1 to 15 aa) was gifted from Dr. Douglas Black.

For tissue immunostaining, mice were anesthetized and transcardially perfused with PBS and then 4% paraformaldehyde. Brain tissues were submerged in 10%, 20%, and 30% sucrose solution for gradient hydration. The resulting tissues were embedded in Optimum cutting temperature (OCT) and cut on a cryostat. The brain sections were blocked in 3% Bovine Serum Albumin (BSA) and then incubated with primary antibody overnight. After 0.5% PBS-T washes, the sections were incubated with Alexa Fluor-conjugated secondary antibodies (Thermo Fisher Scientific). For cultured cell staining, cells were fixed with 4% paraformaldehyde and blocked with blocking buffer. The fixed cells were incubated with primary antibodies overnight. Images were taken by Nikon A1 confocal microscopy.

### Immunoblot, IP, and co-IP/MS

For immunoblot, fresh tissues were homogenized on ice with Radioimmunoprecipitation assay (RIPA) buffer (25 mmol/L Tris–HCl, pH 7.6, 150 mmol/L NaCl, 1% NP-40, 1% sodium deoxycholate, 0.1% SDS) complemented with protease inhibitor cocktail (Roche) and phosphatase inhibitor cocktail (PhosSTOP, Roche). For cell lysate, the cultured cells were washed with PBS first and then lysed with RIPA buffer. The resulting lysates were centrifuged at 12,000 ×*g* for 10 min. Supernatant was boiled with 2× SDS loading buffer for immunoblot loading.

For Immunoprecipitation (IP), mouse cerebellum was homogenized on ice with IP buffer (150 mmol/L KCl, 25 mmol/L Tris, pH 7.4, 5 mmol/L EDTA, 1% Nonidet P-40) complemented with protease and phosphatase inhibitor cocktail (Roche). Cultured cells were washed with ice-cold PBS three times and lysed with IP buffer on ice. Tissue and cell lysates were rotated at 4 °C for 0.5 h and then centrifuged to remove cell debris. The supernatant was collected and incubated with anti-FLAG M2 magnetic beads (M8823, Merck) 4 °C overnight. The magnetic beads were washed with IP buffer twice and Tris buffered saline (TBS) buffer (50 mmol/L Tris–HCl, 150 mmol/L NaCl, pH 7.4) twice. The resulting samples were boiled with 2× SDS loading buffer.

For Mass Spectrometry (MS), samples were loaded on Sodium dodecyl sulfate–polyacrylamide gel electrophoresis (SDS-PAGE) gels for separation, stained with SimplyBlue™ SafeStain (Life technologies), and excised. The gel slices were reduced and in-gel digested with sequencing grade modified trypsin (Promega). The resulting samples were quenched by 10% trifluoroacetic acid and peptides were extracted and dissolved in 0.1% trifluoroacetic acid. For LC-MS/MS, the purified peptides were separated by a C18 column (75 μm inner diameter, 150 mm length, 5 μm, 300 Å) and directly connected with an Orbitrap Fusion Lumos mass spectrometer (Thermo Fisher Scientific). Mobile phase A was an aqueous solution of 0.1% formic acid, and mobile phase B was 0.1% formic acid in acetonitrile. The MS/MS spectra were searched against the Uniport mouse database using Proteome Discoverer (version PD1.4, Thermo Scientific™). The peptide spectrum match (PSM) was calculated by Percolator provided by Proteome Discoverer, and only peptide FDR less than 0.01 was included for further analysis. Peptides only assigned to a given protein group were considered as unique. FDR was also set 0.01 for protein identifications.

### Proteome analysis for potential peptides encoded by DIs and their upstream exons

For proteome analysis, cerebellum was quickly removed and placed in a Dounce homogenizer. Tissues were lysed with 8 mol/L urea in PBS supplemented with protease and phosphatase inhibitor cocktail (Roche) at room temperature. Lysates were centrifuged at 12,000 rpm for 10 min and supernatant was collected. Protein concentration was determined by a BCA Protein Assay Kit (Pierce™). The tryptic peptides were fractionated with a XBridgeTM BEH300 C18 column (Waters, MA). LC-MS/MS analysis was similar to what we described above. To identify potential peptides encoded by DIs, a customized peptide database was generated as previously reported (Wong et al., 2013). Briefly, all DI events were extracted from wildtype and *NMF291*^−/−^ RNA-Seq results. The RNA sequences were *in silico* translated to peptides from 60 nucleotides upstream of DIs, until a PTC is encountered. The MS/MS spectra from each LC–MS/MS run were searched against the customized peptide database by a Sequest HT search engine of Proteome Discoverer, to identify potential peptides encoded by intronic sequences. Trypsin was specified as the proteolytic enzyme, and we allowed up to two missed cleavage sites. PSM was validated using Percolator, and only FDR < 0.01 was considered correct.

### Total RNA extraction, reverse transcription, real-time qPCR, and nuclear and cytoplasmic RNA extraction

Total RNA was extracted with TRIzol reagent (Thermo Fisher Scientific). The extracted RNA was dissolved in nuclease-free water and then treated with DNase (RQ1 RNase-Free DNase, Promega). Reverse transcription was achieved with M-MLV Reverse Transcriptase (Promega). Real-time qPCR was achieved with qPCR SYBR Green Mix (Yeasen).

For nuclear and cytoplasmic RNA extraction (Hwang et al., 2007), N2a cells were placed on ice, washed with ice-cold PBS three times, and then collected by spinning at 1000 rpm for 10 min. The resulting cells were resuspended in 200 μL lysis buffer A (pH 8.0, 10 mmol/L Tris, 140 mmol/L NaCl, 1.5 mmol/L MgCl_2_, 0.5% NP-40, 1 mmol/L DTT, 100 U/mL RNasin), incubated on ice for 5 min, then centrifuged at 1,000 ×*g* for 3 min. The supernatant was collected for cytoplasmic RNA extraction. The pellets were further washed twice with the lysis buffer A and once with the lysis buffer A complemented with 1% Tween-40 and 0.5% deoxycholic acid. The resulting pellets were resuspended in TRIzol for nuclear extraction.

### RNA-Seq

Total RNA was dissolved in nuclease-free water, treated with DNase (Ambion, Thermo Fisher Scientific), and quantified by a Qubit RNA Assay Kit (Thermo Fisher Scientific). Agilent 2100 Bioanalyzer (Agilent Technologies) was employed to check the quality of RNA. Total RNA (3 μg) was applied for poly(A) mRNA purification by using oligo-d(T) magnetic beads (S1419S, NEB). RNA fragmentation, cDNA synthesis, terminal repair, A-tailing, and adapter ligation were performed using an RNA library prep kit (E7530L, NEB). DNA products were cleaned using AMPure XP beads (Beckman). Library quality was checked by Agilent 2100 Bioanalyzer and quantified by real-time PCR. Sequencing was performed on the Illumina HiSeq platform.

### IR analysis

The quality of RNA-Seq reads was assessed by FastQC. The adaptors and low-quality reads were removed by Cutadapt to obtain clean reads. The resulting reads were aligned to mouse genome mm10 using HISAT2 (Kim et al., 2019). IR was analyzed as previously reported with slight modifications (Jia et al., 2012; Wong et al., 2013; Braunschweig et al., 2014). In brief, every intron in the genome was considered as a potential retained intron, while only introns with both flanking exons covered by the aligned reads were included for further analysis. To avoid the influence of small non-coding RNAs and unknown exons located inside of the introns and low mappability regions in large introns, including high GC regions and repetitive sequence, only reads covering exon–intron junctions and exon–exon junctions were used to calculate the IRI (intron retention index). IRI for 5ʹ SS and 3ʹ SS were calculated separately. We considered the exon–intron junction reads as intronic reads, and the sum of exon–intron and exon–exon junction reads as total reads. IRI = (5ʹ SS intronic reads/ 5ʹ SS total reads + 3ʹ SS intronic reads/ 3ʹ SS total reads)/2. For RNA-seq data, IRI > 0.1 and intron coverage > 0.9 were set to identify reliable IR events. For the comparison of differential intron usage, DEXSeq was employed for the statistical analysis, which offers reliable control of *P*_adj_ by estimation of dispersion for each counting bin with generalized linear models (Anders et al., 2012).

### Heatmap, GO analysis, and conservation analysis

Heatmaps of Z-scores were plotted using Heatmap.2 of the R package “gplots.” To compare reliable DI events across different conditions, only events with IRI > 0.1 were included in the analysis. For GO enrichment analysis, the PANTHER Classification System was used to test the overrepresentation of genes (Mi et al., 2013). Statistical overrepresentation testing was performed for the GO biological process complete category and the enriched GO terms were ranked by fold-enrichment and FDR and the redundant GO terms were manually removed. To determine whether DIs are conserved between human and mouse, intron coordinate conversions were performed using UCSC LiftOver (from mm10 to hg38). The highly conserved introns (Liftover remap ratio > 90% between human and mouse) were included for the conservation comparison (*P* values were calculated with two-sided proportion tests).

### Nanopore full-length mRNA sequencing

Mouse cerebellar total RNA was extracted using TRIzol and treated with DNase. RNA quality was assessed using an Agilent 2100 Bioanalyzer to ensure the integrity of RNA. Total RNA (1 μg) was used for cDNA library construction, following the protocols suggested by Oxford Nanopore Technologies (ONT). Reverse transcription and strand-switching were performed using a cDNA-PCR Sequencing Kit (SQK-PCS109, ONT). cDNA was PCR amplified for 14 cycles by using LongAmp Taq (NEB). ONT adaptor ligation was performed using T4 DNA ligase (NEB). The resulting DNA was purified by Agencourt XP beads. The final libraries were added to FLO-MIN109 flowcells (ONT), sequenced using PromethION platform. Raw ONT reads were filtered by read quality score (>7) and minimal reads length (>500 bp). Clean ONT reads were aligned to mouse genome reference mm10. Aligned reads were converted to bam files, sorted, and indexed by SAMtools (Li et al., 2009). Reliable full-length transcripts were filtered to contain 5ʹUTR and 3ʹUTR regions by using BEDTools (Quinlan, 2014). To identify reliable DIs, minimal IRI was set as 0.05 with intronic region coverage larger than 0.9.

### Native RIP-seq

Native RIP-seq was performed as previously reported with minor modifications (Rinn et al., 2007). For mouse tissues, mouse cerebellum was removed and placed in a Dounce homogenizer on ice. Lysis was in RIP buffer (150 mmol/L KCl, 25 mmol/L Tris, pH 7.4, 5 mmol/L EDTA, 0.5 mmol/L DTT, 0.5% Nonidet P-40) complemented with protease and phosphatase inhibitor cocktail (Roche) and 100 U/mL RNasin (Promega). For cultured cells, cells were washed with ice-cold PBS three times and lysed in RIP buffer on ice. Tissue and cell lysates went through a 23-gauge needle, rotated at 4°C for 0.5 h, and centrifuged to remove cell debris. The resulting supernatant was incubated with anti-FLAG M2 magnetic beads (M8823, Merck). After incubation for 12 h at 4°C, M2 beads were washed with RIP buffer twice and TBS buffer twice. Elution was achieved by using 250 μg/mL 3× Flag peptides (F4799, Sigma). The resulting eluates were resuspended in TRIzol for the following RNA extraction. Smart-seq2 was performed following a standard protocol as previously described (Picelli et al., 2014). The amplified DNA (40 ng) was fragmented into ~350 bp fragments using a Bioruptor® Sonication System (Diagenode Inc.). Illumina library construction was performed and qualified libraries were sequenced on the Illumina HiSeq platform. As described above, we used exon–intron junction and exon–exon junction reads to calculate IRI. For ratio comparison of IRI, IRI > 0.1 and intron coverage > 0.8 were set to identify reliable IR events.

### CLIP analysis

The RNPS1-GFP iCLIP and SF3a60 eCLIP were performed by Hauer et al., (2016) and Van Nostrand et al., (2020a, b) previously, and the raw data were downloaded from ArrayExpress and ENCODE (Consortium, 2012; Athar et al., 2019; Zhang et al., 2020). The CLIP and corresponding RNA-Seq reads were aligned to the human reference genome hg19 by STAR v2.7 (Dobin et al., 2013). The second read (R2) in each eCLIP read pair was extracted by SAMtools for downstream analysis, as described in the eCLIP-seq processing pipeline (Van Nostrand et al., 2016). To generate a CLIP read density plot, biological replicates were merged for combined reads count. Introns were grouped based on IR analysis in CLIP-related RNA-Seq data. DIs with high confidence were filtered by IRI > 0.2 and intron region coverage > 0.95, while efficiently spliced introns were filtered by IRI < 0.05. CLIP read coverage of introns and intron flanking exons were calculated using BEDTools (Quinlan, 2014), then read coverage was summed and normalized according to their relative position to 5ʹ SS and 3ʹ SS. The final read density was plotted using R (version 3.4.3). CLIP peak calling was achieved with CLIPper (CLIP peak enrichment recognition) by the default parameters (Lovci et al., 2013). Binding stringency was ranked by the *P*-value calculated by CLIPper.

## Supplementary Material

pwac008_suppl_Supplementary_Material

## Data Availability

The RNA-seq datasets have been deposited in the NCBI’s Sequence Read Archive, which can be accessed by the BioProject ID PRJNA824597. Other data and materials supporting the findings of this study are available from the corresponding author upon request.

## Abbreviations

AS: alternative splicing
ASAP: apoptosis and splicing-associated protein
B^act^: active spliceosome
BS: branch site
CHX: cycloheximide
cKO: conditional knockout
CLIP: cross-linking and immunoprecipitation
DI: detained intron
EGL: external granule layer
EJC: exon junction complex
ENU: N-ethyl-N-nitrosourea
FHA: forkhead-associated
GO: gene ontology
IDTs: intron-detaining transcripts
IGL: internal granule layer
IGV: Integrative Genomics Viewer
IR: intron retention
KI: knockin
KO: knockout
ML: molecular layer
NMD: nonsense-mediated decay
PTC: premature termination codon
RES: REtention and Splicing complexes
RIP: RNA immunoprecipitation
RNPS1: RNA binding protein with serine rich domain 1
RRM: RNA recognition motif
SNIP1: Smad nuclear interacting protein 1
snRNA: small nuclear RNA
snRNPs: small nuclear ribonucleoprotein particles
SS: splice site
