# Supplementary material for: A molecular brake that modulates spliceosome pausing at detained introns contributes to neurodegeneration
Source: Protein Cell. 2022 Nov 11;14(5):318–36. doi: 10.1093/procel/pwac008 (PMC10166177; doi:10.1093/procel/pwac008)
Supplement: pwac008_suppl_Supplementary_Material [file pwac008_suppl_supplementary_material.pdf]

Supplementary Information for

A molecular brake that modulates spliceosome pausing at detained introns contributes to neurodegeneration

Dawei Meng, Qian Zheng, Xue Zhang, Xuejiao Piao, Li Luo, and Yichang Jia*

*Corresponding author.
Email: yichangjia@tsinghua.edu.cn

This PDF file includes:

Figs. S1 to S10
Tables S1 to S3
Legend for Movie S1

Other Supplementary Materials for this manuscript include the following:

Movie S1

Supplementary Figures

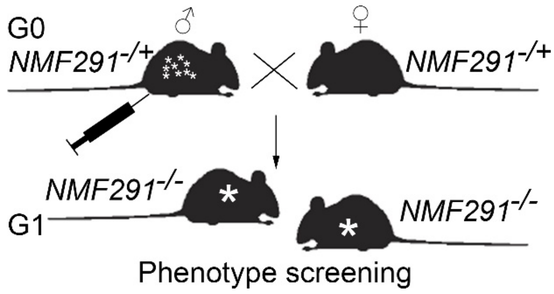

Figure S1. An ENU-induced mutagenesis screening for dominant modifier(s) that rescue(s) the *NMF291*$^{-/-}$ phenotypes.
G0 male *NMF291*$^{-/+}$ mice were injected with ENU (N-ethyl-N-nitrosourea) once a week for 3 weeks. After their fertilities recovered, they were bred to ENU-untreated female *NMF291*$^{-/+}$ mice. The G1 *NMF291*$^{-/-}$ mice carrying less ataxia phenotype and improved lifespan were crossed to ENU-untreated *NMF291*$^{-/+}$ mice to determine the family pedigree.

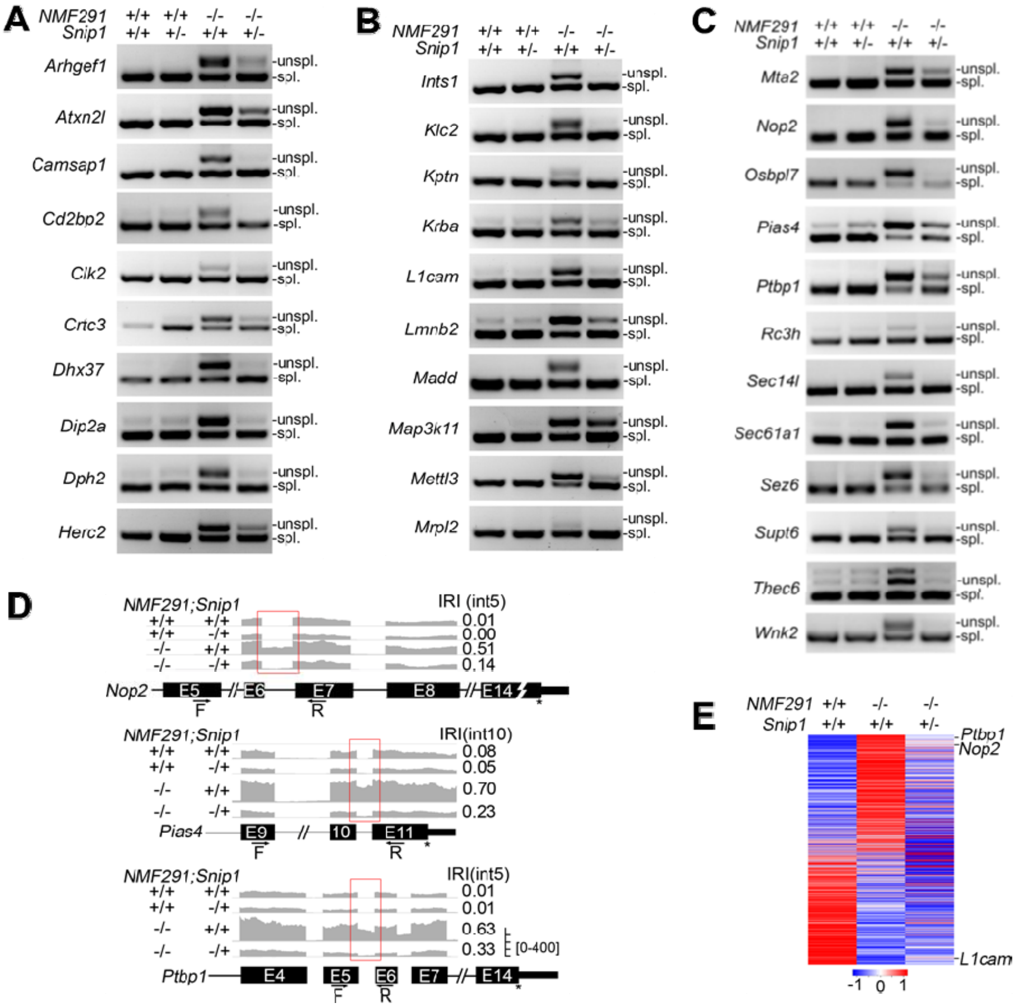

Figure S2. Haploinsufficiency of *Snip1* partially rescues the IRs and their corresponding gene expressions.

(A-C) Validation of the IRs rescued by *Snip1*$^{-/+}$ by RT-PCR. Cerebella were harvested at one month of age with the indicated genotypes. Unspl., unspliced transcripts; spl., spliced transcripts.

(D) The representative IRs visualized by IGV. Primers used for the RT-PCR validation in (C) were illustrated. F, forward primer; R, reverse primer.

(E) Corresponding genes (1455) of the rescued IRs (1961) shown in Figure 2C were included for expression analysis. The Z-score was used to normalize expression level in each row.

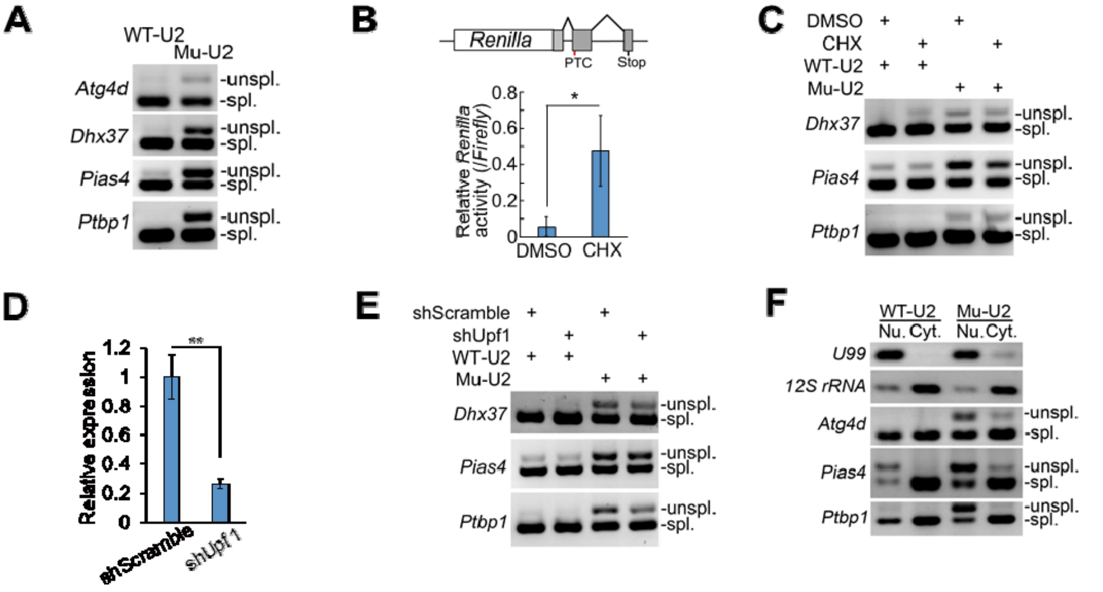

Figure S3. Intron-containing transcripts overrepresented in *NMF291* mutant cerebellum are likely IDTs.

(A) The DIs amplified by Mu-U2 but not WT-U2 in N2a cells.

(B) Previously described a NMD reporter (PMID: 1693475) in which *Renilla* luciferase contains a PTC in the second last exon. Application of CHX significantly inhibited NMD measured by relative *Renilla* activity. The *Firefly* luciferase was used to normalize transfection efficiency.

(C) The intron-containing transcripts were insensitive to application of CHX in culture medium of N2a cells expressing WT-U2 or Mu-U2.

(D and E) N2a cells were infected with *Upf1* and scrambled shRNA (MissionRNAi, Sigma). The relative expression level of *Upf1* measured by quantitative-PCR (D). The intron-containing transcripts were detected by RT-PCR (E).

(F) The nuclear and cytosolic fractions evidenced by the enrichments of *U99* and *12s rRNA*, respectively. Nu., nucleus; Cyt., cytosol.

In B and D, the values are presented as mean ± SEM, *p<0.05, **p<0.01, n = 4, t-test, SPSS. In A, C, E, and F, unspl., unspliced transcripts; spl., spliced transcripts.

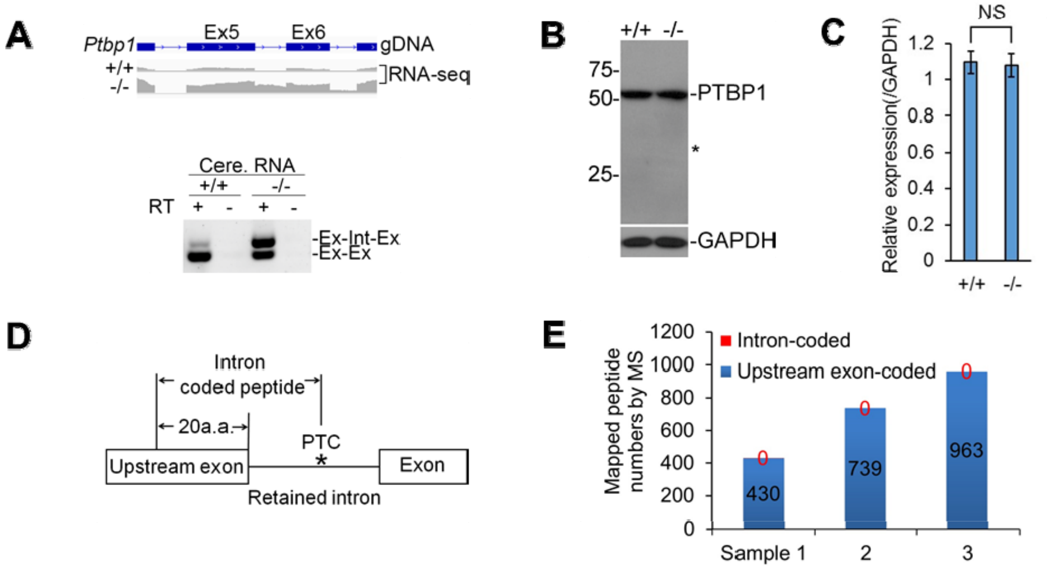

Figure S4. DIs are likely not code proteins.

(A) Detained intron 5 of *Ptbp1* was evidenced by both RNA-seq (upper) and RT-PCR (lower) in wildtype (+/+) and *NMF291* mutant (-/-) cerebella.

(B and C) The expression of PTBP1 by immunoblot. GAPDH served as loading control. Asterisk marked the corresponding molecular weight of PTBP1 supposedly coded by the intron 5-containing transcript.

(D) A pipeline combines RNA-seq and MS (mass spectrometry) for seeking intron-coded peptide. Based on the DIs we identified in the *NMF291*$^{-/-}$ cerebellum, we generated customized peptide database, which are coded by the DIs and their upstream exons.

(E) Protein lysates from +/+ (n = 3) and *NMF291*$^{-/-}$ mutant (n = 3) cerebella were applied for MS. The MS hits of our customized peptide database from one +/+ and mutant pair were pooled and summarized as one smaple. Mouse, one month of age.

In A, unspl., unspliced transcripts; spl., spliced transcripts. In C, the values are presented as mean ± SEM. NS, no statistical significance (n = 3, t-test, SPSS).

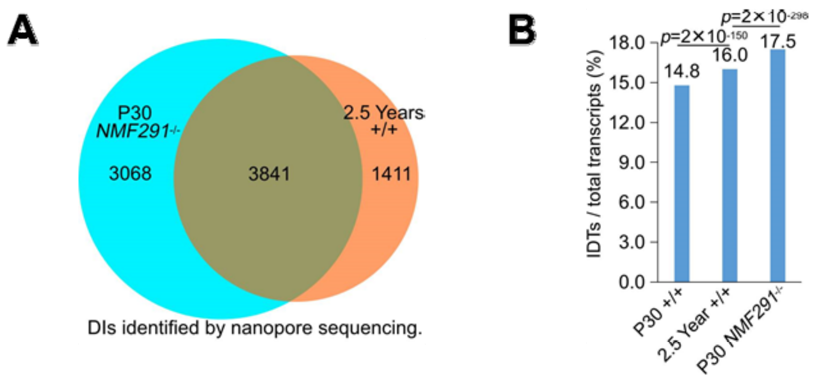

Figure S5. IDTs overrepresented in the *NMF291* mutant mouse are also accumulated in aged cerebellum, revealed by nanopore sequencing.

(A) The majority of DIs accumulated in aged wildtype cerebellum are overlapped with that of the P30 *NMF291*$^{-/-}$ cerebellum.

(B) Percentage of IDTs in young (P30), aged (2.5 years), and P30 *NMF291*$^{-/-}$ cerebella. All full-length nanopore reads containing 5' UTR and 3' UTR were included for the calculation. p values correspond to two-sided proportion tests.

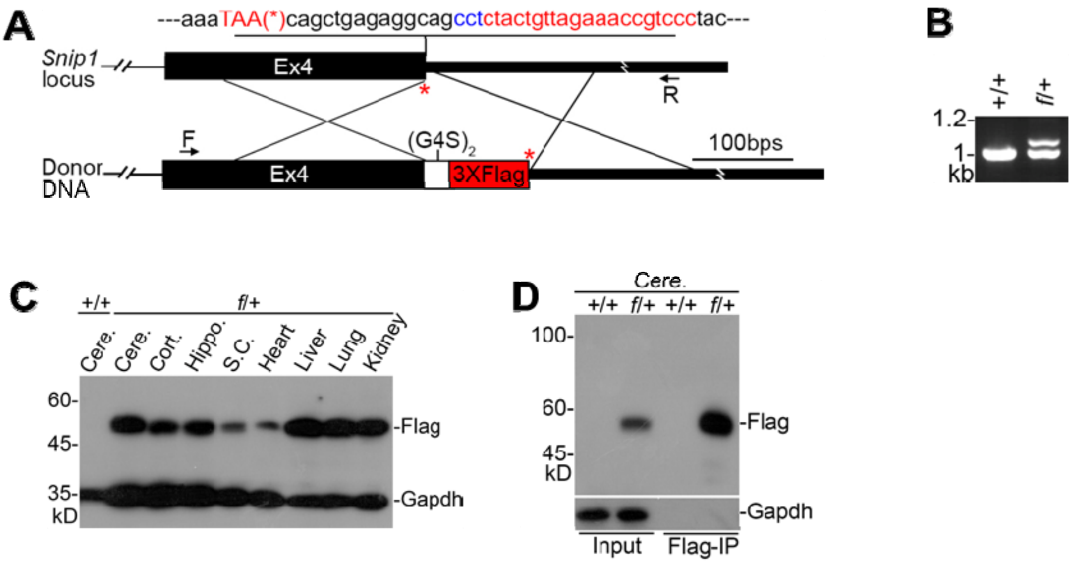

Figure S6. Generation of *Snip1-Flag* mouse.

(A) Crispr/Cas9-based *Snip1-Flag* knockin (KI) design. The mouse *Snip1* locus and *Snip1* last coding exon (exon 4, Ex4) are shown (Upper). The stop codon (TAA) labeled with an asterisk (*); the target sequences of gRNA and PAM site (NGG) labeled in red and blue, respectively. In the donor DNA, a 3-time *Flag* tag was fused with *Snip1* last coding exon. A linker placed between Ex4 and the *Flag* sequences, which codes 2-time G4S $(G4S)_2$.

(B) Genomic DNA PCR confirmed the right KI. Primers for the PCR were labeled in (A) by arrows. F, forward; R, reverse. *f*/+, mouse heterozygous for *Snip1-Flag* KI.

(C) SNIP1-Flag is ubiquitously expressed in various adult mouse tissues (mouse age, one month).

(D) Flag-IP was performed with cerebellar protein lysates from *Snip1-Flag* KI mouse.

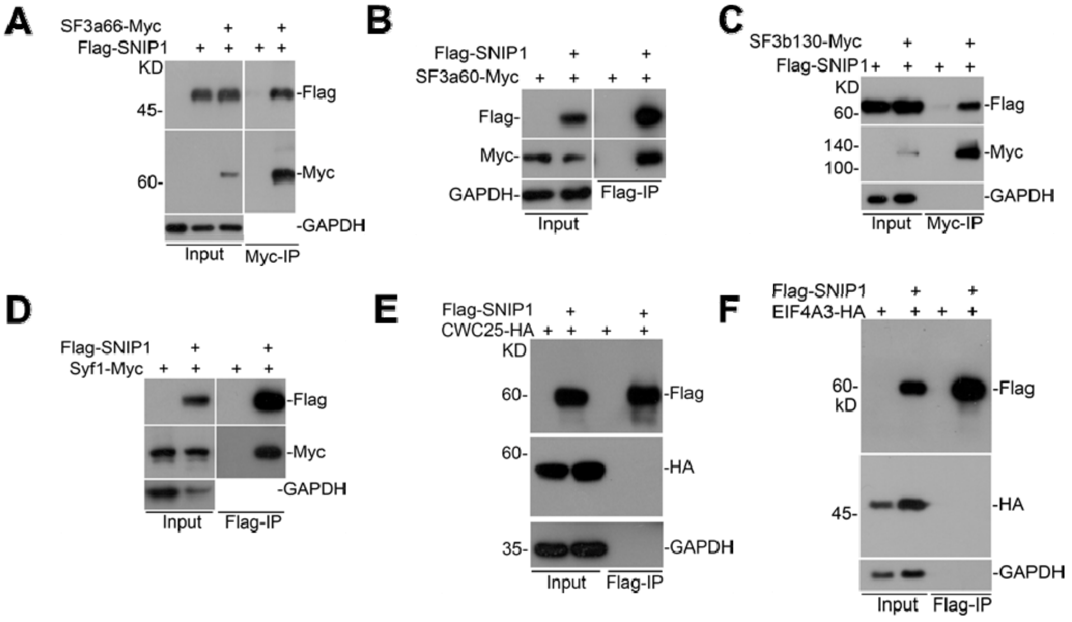

Figure S7. SNIP1-interacting partners are protein components found in B^{act} but not B*.

(A-F) Interactions between SNIP1 and protein components found in B^{act} (SF3a66, SF3a60, SF3b130, and Syf1) but not those in B* (CWC25 and EIF4A3) were validated in N2a cells expressing the indicated tagged proteins.

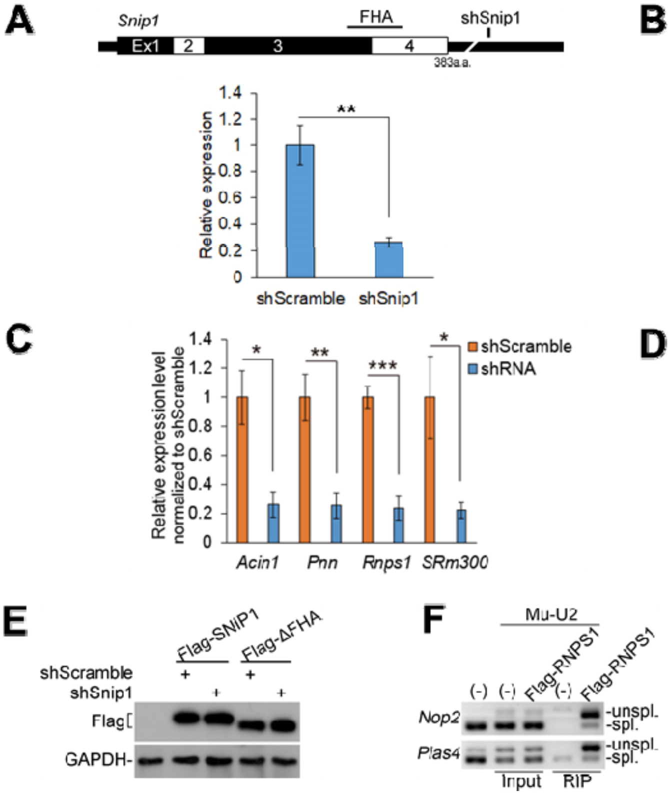

Figure S8. SNIP1 and RNPS1 function as a molecular brake to pause spliceosome at DIs.
(A) Knockdown of *Snip1* in N2a cells by lentiviral shRNA (shSnip1). Note the target sequence of shSnip1 located in the 3'UTR (upper). The relative expression level of *Snip1* measured by quantitative-PCR (lower). The scrambled shRNA served as a control.
(B) Knockdown of *Snip1* reduced DIs amplified by Mu-U2. The shRNA infected N2a cells were transfected with WT- and Mu-U2 expression plasmids, respectively. Scrambled shRNA infection served as a control.
(C) Knockdown of genes encoding peripheral EJC components (*Acin1*, *Pnn*, and *Rnps1*) and a spicing factor (*SRm300*) measured by quantitative-PCR.
(D) The interactions of SNIP1, PNN, and RNPS1 were evidenced by co-IP in N2a cells expressing PNN-HA, Flag-SNIP1, and RNPS1-Myc, simultaneously.
(E) The shSnip1 targets 3'-UTR of endogenous *Snip1*, which does not affect the expression of exogenous full-length and ΔFHA SNIP1 in N2a cells.
(F) Flag-RIP was performed with protein lysates from N2a cells infected with Flag-RNPS1 and transfected with Mu-U2 expression plasmid.
In A and C, the values are presented as mean ± SEM, n = 4. *p<0.05, **p<0.01, ***p<0.001, N.S., no statistical significance, t-test or ANOVA, SPSS.
In B and F, DIs were measured by RT-PCR. Unspl., unspliced transcripts; spl., spliced transcripts.

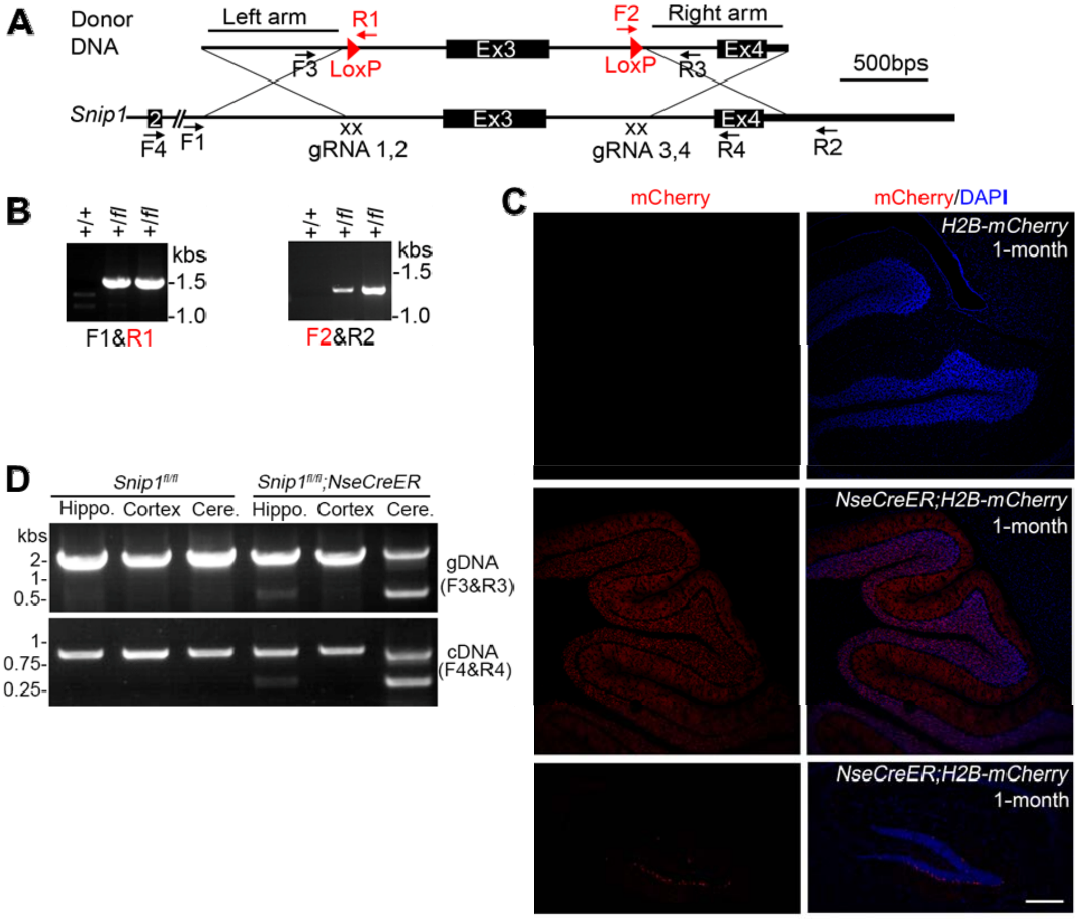

Figure S9. Generation of *Snip1* cKO mouse.

(A) Crispr/Cas9-based *Snip1* cKO design. Genomic structure of mouse *Snip1* is illustrated. Homology arms (Left and Right) in donor DNA were labeled. Four gRNAs (2 gRNAs each side) flanking the *Snip1* exon3 (Ex3) were employed to increase homologous recombination. Primers for genotyping and detecting Cre-induced Ex3 deletion were labeled. Primers (R1 and F2) covering LoxP sites were labeled in red. F1 and R2 are located outside the homology arms.

(B) Genomic PCR detected right LoxP site insertions in mice heterozygous for the floxed allele (+/*fl*). Primers used were labeled in (A).

(C) Confirmation of the *NseCreER* expression by a previously described Cre-reporter, *H2B mCherry*. The *NseCreER* were predominantly expressed in cerebellar granule cells and a few expressed in the granule neurons in hippocampal dentate gyrus.

(D) Cre-dependent Ex3 deletion was detected at both genomic DNA (gDNA) and RNA levels in cerebellum and hippocampus but not in cortex in *Snip1*$^{fl/fl}$;*NseCreER* mouse. *Snip1*$^{fl/fl}$ mouse served as negative control. Tamoxifen injections on P3, 4, and 5; DNA/RNA harvest on P7.

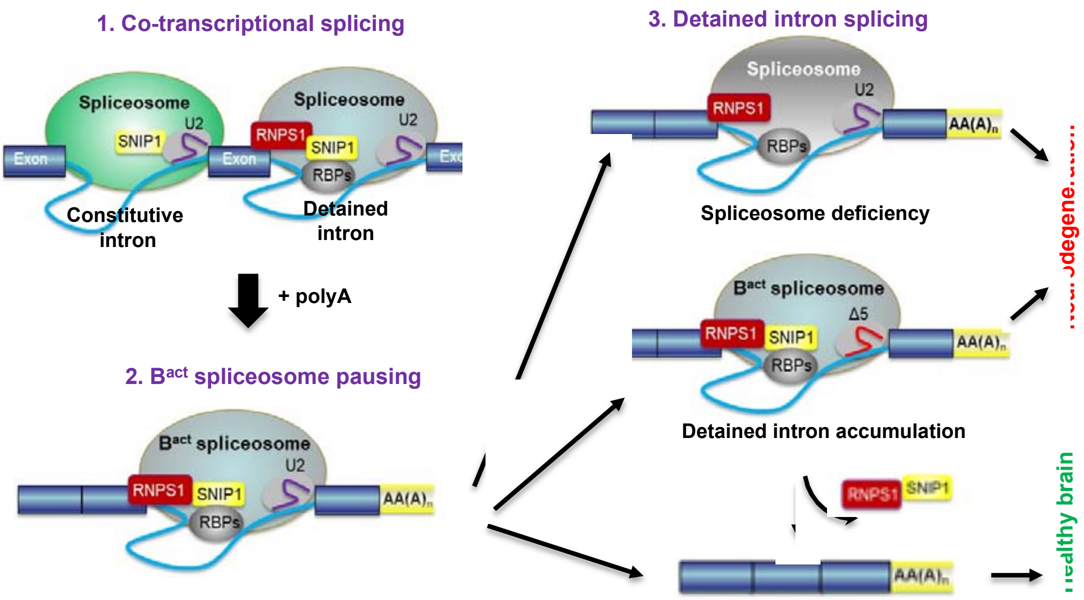

Figure S10. Our working model for post-transcriptional spliceosome pausing at highly regulated DIs and its contribution to neurodegeneration.

We suggest that DI splicing is paused at B^{act} state, an active spliceosome but not catalytically primed, and SNIP1 and RNPS1 function as a molecular brake for the spliceosome pausing. The whole process can be divided into several steps. 1) Spliceosome loaded at both constitutive and detained introns. After constitutive introns are spliced, pre-mRNAs are polyadenylated to complete the co-transcriptional splicing. 2) The splicing of DIs is paused at B^{act} state (spliceosome pausing), which is mediated by SNIP1/RNPS1 containing complex. SNIP1, RNPS1 and B^{act} component preferentially dock at DIs, forming a molecular brake to modulate spliceosome pausing. RNPS1 recognizes the DIs and its neighboring sequences through its RRM (RNA recognition motif). Interaction between SNIP1 and RNPS1 is mediated by SNIP1 FHA (forkhead-associated) domain and RNPS1 S (serine-rich) domain. 3) The paused spliceosome is resumed, and DIs are spliced. The completely spliced transcripts are exported to cytosol for protein translation. Mutant (the *NMF291*$^{-/-}$) or dysfunctional (*Snip1*$^{-/-}$) spliceosome decreases splicing efficiency of DIs but has little effect on that of constitutively-spliced introns. That leads to accumulation of DIs, which in turn worsens DI splicing by sequester of spliceosome pausing complex and depletion of the available complex components and further damages the corresponding gene functions, and eventually causes neurodegeneration. Partial loss of SNIP1 or RNPS1 rescues intron detentions caused by expression of mutant U2, probably through releasing the molecular brake.

Table S1. Modifier candidates for the *NMF291* phenotypes.

Gene	Position	SNV	Category	Rescue	No rescue
Dnajc1	chr2:18284715	T/C	non-synonymous	1/2	2/2
Fam193a	chr5:34436543	T/C	non-synonymous	1/2	1/2
Ctr9	chr7:111043186	A/G	non-synonymous	2/3	1/3
Gtf3c1	chr7:125646516	G/A	non-synonymous	2/2	1/2
Nlrp1b	chr11:71228365	C/T	non-synonymous	2/2	1/2
Pcdhac1	chr18:37090163	T/A	non-synonymous	1/3	1/3
Ablim1	chr19:57061311	A/T	Stop-gain	2/2	2/2
Frem1	chr4:82914625	T/A	Stop-gain	6/7	2/5
Gm12794	chr4:101941270	T/G	non-synonymous	18/18	1/14
Ptch2	chr4:117114863	T/A	3'UTR	8/8	1/9
Snip1	chr4:125068193	A/G	5'SS/splicing	27/27	0/19
Aunip	chr4:134523450	A/G	synonymous	8/9	0/9
Kif17	chr4:138291508	T/A	non-synonymous	7/8	0/10

Note: The modifier candidates were identified by an exome capture as previously described. The ENU-induced *Snip1* mutation was co-segregated with the rescued phenotype among the candidates we examined, part of which are shown here. The mouse GRCm38/mm10 built was used as the reference genome. SNV, single nucleotide variant. Red, genotypes did not meet the expected phenotypes; green, genotypes agreed with the phenotypes.

Table S2. The abnormal Mendelian ratio seen in the progenies from $Snlp1^{M/+}$ and $Snlp1^{-/+}$ intercrosses.

	$Snip1^{+/+}$	$Snip1^{M/+}$	$Snip1^{M/M}$
Expected segregation ratio	11	22	11
Observed segregation ratio	10	34	0

Note: The Chi-square value was equal to 17.64 ($p < 0.001$)

	$Snip1^{+/+}$	$Snip1^{-/+}$	$Snip1^{-/-}$
Expected segregation ratio	11.75	23.5	11.75
Observed segregation ratio	16	31	0

Note: The Chi-square value was equal to 15.68 ($p < 0.001$)

Table S3. SNIP1-interacting protein partners identified by Flag co-IP/MS.

Gene name	FLAG-SNIP1-1	(-)	FLAG-SNIP1-2	(-)	Annotation	Reference
Snip1	1655.64	0	1926.17	0		
Clasrp/SFRS16	94.2	0	115.1	0	SR protein function largely unknown.	PMID:12169693
Prpf8/prp8	65.32	0	123.94	0	Scaffolding protein at the catalytic core of the spliceosome.	PMID: 15840809
Snrnp200/Brr2	68.75	0	70.59	0	Helicase and one of the components of tri-snRNPs to initiate spliceosome activation by unwinding the U4/U6 snRNA helices.	PMID: 28781166
Acin1	63.47	0	70.5	0	SR protein and component of ASAP complex.	PMID: 22388736 PMID: 20966198 PMID:16314458
Sf3b3/Sf3b130	62.1	0	41.49	0	SF3b complex component and displacement from the spliceosome initiates the first step of the splicing reaction	PMID: 29360106
Psmd14	2.2	0	98.2	0	Proteasome subunit	
Rnps1	20.43	0	79.25	0	SR protein and component of ASAP complex	PMID: 22388736 PMID: 20966198
Uqcrc2	69.83	0	25.71	0	Mitochondrial ubiquinol-cytochrome c reductase core protein II	
Srrm2/SRm300/Cwc21	56.6	0	37.35	0	SR protein and component of NTC	PMID: 25740649 PMID: 19854871
Zc3h14	39.36	0	53.77	0	Polyadenosine RNA-binding protein, RNA quality control	PMID: 27563065
Cfap20	22.11	0	45.48	0	Cilia and flagella associated protein 20	
Srsf7/9G8	3.03	0	52.06	0	SR protein, hypophosphorylation facilitate mRNA export	PMID: 15210956
Pnn	24.68	0	30.18	0	Component of NTC	PMID: 22388736
Sf3b2/Sf3b145	26.92	0	24.37	0	SF3b complex component and displacement from the spliceosome initiates the first step of the splicing reaction	PMID: 29360106
Luc7l2	15.72	0	31.8	0	LUC7L2, a mammalian homolog of a yeast protein involved in recognition of non-consensus splice donor sites.	
Cdc5l/Cef1/cdc5	31.39	0	11.95	0	Key component of NTC	PMID: 12411573
Bclaf1	18.19	0	25.14	0	Bcl-2-associated transcription factor 1, mRNA nuclear export	PMID: 22833098
Xab2/Syf1	22.46	0	20.17	0	Interact with Debranching enzyme 1 and AQR Intron Large Complex	PMID: 17981804 PMID: 25671812
Rack1	12.95	0	22	0	Ribosome subunit, involved in ribosome-Associated Quality Control Function	PMID: 28132843
Psma4	6.5	0	22.52	0	Proteasome subunit	
Zc3h18	18.63	0	7.56	0	RNA export	PMID: 24782531
Tpi1	20.93	0	3.54	0	Triosephosphate isomerase	
Gm9774	6.31	0	15.91	0	N.A.	
Nif3l1	7.25	0	14.97	0	Function unknown	
Eif2s1/Eif2a	2.83	0	18.9	0	Catalyzes an early regulated step of protein synthesis initiation	
Sf3a3/Prp9/Sf3a60	11.81	0	9.63	0	SF3a complex component and displacement from the spliceosome initiates the first step of the splicing reaction	PMID: 22314233
2310022A10Rik	13.67	0	6.44	0	N.A.	
Sf3b1/Sf3b155	7.17	0	1.82	0	SF3b complex component and displacement from the spliceosome initiates the first step of the splicing reaction	PMID: 29360106
Aqr	4.37	0	4.27	0	Component of NTR	
Sf3a1/Prp21/Sf3a120	69.35	13.46	69.42	0	SF3a complex component and displacement from the spliceosome initiates the first step of the splicing reaction	PMID: 29360106
Prpf19/Prp19	60.14	0	75	2.03	Key component of NTC	PMID: 12411573
Snw1/SKIP	64.31	0	65.08	2.33	Component of NTC	PMID: 25450007
Eftud2/Snu114	117.21	9.04	101.14	5.15	Component of NTC and U5 snRNPs	PMID: 25740649 PMID: 19854871

Note: Flag co-IP was performed with cell lysates from N2a cells stably expressing Flag-SNIP1 and then the co-IP products were applied for MS. Naïve N2a cells were employed as negative control (-). We only included SNIP1 interacting candidates that appeared at least in two out of three replicates in Flag-SNIP1 group but not in control group or their average MS scores in Flag-SNIP1 group is 5 times higher than that of control group. In addition, we filtered out the hits their MS score < 2. Brief function annotation of each candidate was included in this table.

Legend for Movie S1 (separate file).

Of these two littermates (4-month old, *NMF291*$^{-/-}$), one carried a modifier, a G to A mutation that hits the splice site of *Snip1* exon2 (Figure 1B), partially rescued the severe ataxia phenotype caused by the *NMF291* mutation.
